# A deep-learning-based workflow to deal with the defocusing problem in high-throughput experiments

**DOI:** 10.1016/j.bioactmat.2021.09.018

**Published:** 2021-09-16

**Authors:** Yunfan Xue, Honglin Qian, Xu Li, Jing Wang, Kefeng Ren, Jian Ji

**Affiliations:** MOE Key Laboratory of Macromolecule Synthesis and Functionalization, Department of Polymer Science and Engineering, Zhejiang University, Hangzhou, 310027, PR China

**Keywords:** High-throughput, Deep learning, Cell imaging, Refocusing, Microscopy

## Abstract

The increasing throughput of experiments in biomaterials research makes automatic techniques more and more necessary. Among all the characterization methods, microscopy makes fundamental contributions to biomaterials science where precisely focused images are the basis of related research. Although automatic focusing has been widely applied in all kinds of microscopes, defocused images can still be acquired now and then due to factors including background noises of materials and mechanical errors. Herein, we present a deep-learning-based method for the automatic sorting and reconstruction of defocused cell images. First, the defocusing problem is illustrated on a high-throughput cell microarray. Then, a comprehensive dataset of phase-contrast images captured from varied conditions containing multiple cell types, magnifications, and substrate materials is prepared to establish and test our method. We obtain high accuracy of over 0.993 on the dataset using a simple network architecture that requires less than half of the training time compared with the classical ResNetV2 architecture. Moreover, the subcellular-level reconstruction of heavily defocused cell images is achieved with another architecture. The applicability of the established workflow in practice is finally demonstrated on the high-throughput cell microarray. The intelligent workflow does not require a priori knowledge of focusing algorithms, possessing widespread application value in cell experiments concerning high-throughput or time-lapse imaging.

## Highlights

• An automatic workflow for the sorting and reconstruction of defocused images.
• Representative data containing varied cells, substrate materials, and magnifications.
• Effectiveness of the workflow in high-throughput experiments.
• Publicly accessible image datasets and codes.

## Graphical abstract

**Figure:**
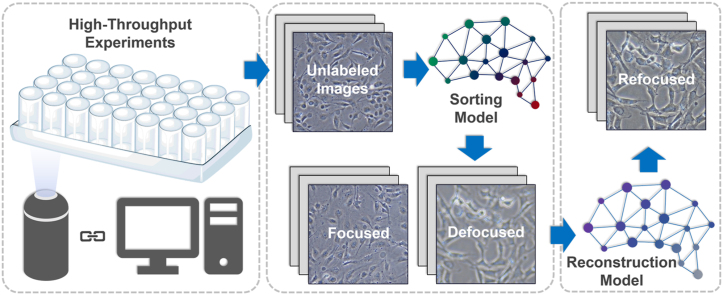

## 1 Introduction

High-throughput techniques are turning impractical experiments into routines. A variety of studies [[1], [2], [3], [4], [5], [6], [7], [8], [9]] has demonstrated the potential of high-throughput experiments in biomaterials science, where the optical microscope is one of the most efficient and accessible instruments for the characterization of cells. Focusing, throughout the imaging process in microscopy, is a crucial step to high-quality data. Automatic cell imaging nowadays mainly relies on specific focusing algorithms [[10], [11], [12]] which are normally widely applicable but may not be precise enough on some cellular images with background noises which are, however, very common for biomaterials (the word noises here refer to entities such as micro air bubbles in hydrogel instead of the hot pixel noises caused by the light sensor in general). Additionally, with the accidental errors caused by mechanical equipment or software, defocused cell images can still be acquired from time to time. In common experiments, these images can be removed manually and re-taken immediately, but the efficiency of manual operation is unacceptable in high-throughput experiments. In addition, there is inherently no chance for re-taking in automatic time-lapse imaging experiments once the time point is missed. Accordingly, achieving the automatic sorting and reconstruction of defocused cell images is of great significance.

Deep learning based on convolutional neural networks (CNNs) is a rising tool in the biomedical field. The inputs of a CNN model are usually various biological images from single-cell images to whole-slide tissue images. Through a series of black-box operations, the input image can be converted to another image or specific values representing categories or biological indexes. The potential of CNN has been illustrated not only in image-to-category tasks [[13], [14], [15], [16], [17], [18], [19], [20], [21], [22]] (e.g., classification of stem cell states [14,16]) but also in image-to-image tasks [[23], [24], [25], [26], [27], [28], [29], [30], [31], [32]] (e.g., generating virtual stained images from unlabeled cell images [24,25,30,31]). These two kinds of tasks fit well with our targets of sorting and reconstruction of defocused images. Furthermore, as a black-box process, deep learning does not require users of a priori knowledge of imaging or optics, which is an important advantage for wide-range applications. Based on its superiorities, here, we present a deep-learning-based workflow to achieve the fast and high-accuracy sorting and the subcellular-level reconstruction of defocused phase-contrast cell images to deal with the defocusing problem in high-throughput experiments.

In this study, we first prepared a high-throughput cell microarray to illustrate the defocusing problem in practice. Then, to establish the deep learning models in the workflow, a comprehensive dataset containing images of two magnifications, three types of cells, and three substrate materials was prepared. On the dataset, we compared the performance of a ResNet50V2 [33] architecture with our self-defined convolutional neural network (SDCNN) with a very simple architecture. We found that with a proper approach to adjust image resolution, the time required for the training process can be significantly compressed without the sacrifice of classification accuracy. The SDCNN model achieved an accuracy of more than 0.993 and required only approximately 0.5 h for the training process on a dataset containing 10,000 images which was less than half of the time needed for ResNet50V2. A modified UNet [34] architecture was used in the reconstruction of defocused images and it was shown that subcellular structures could be precisely reconstructed even when most of them could not be sensed by human eyes in images before reconstruction. The model also performed great generalization ability that it could be directly applied to images of new cell types and substrates without further training. Moreover, the complete workflow had practical performance in images collected from the high-throughput cell microarray, where all the defocused images were found and reconstructed, showing its potential in experiments concerning automatic cell imaging.

## 2 Results

### 2.1 The defocusing problem in high-throughput experiments

**Fig. 1:**
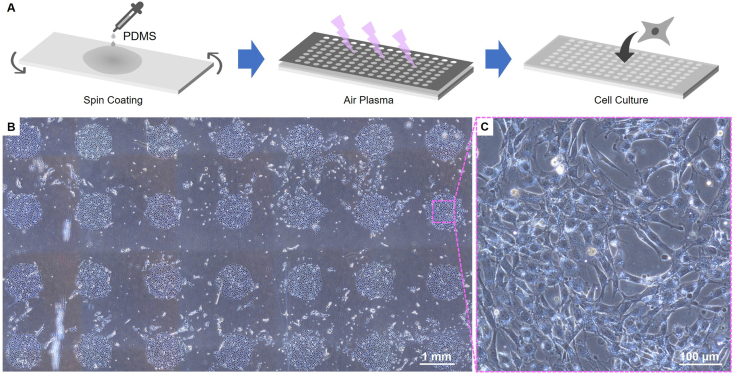
The high-throughput cell microarray. (**A**) The preparation process of the high-throughput microarray. The prepolymer of PDMS was spin-coated on a glass slide and then solidified. A metal mask was covered on the PDMS substrate during the air plasma treatment to obtain a microarray for cell adhesion. The prepared chip was then used for the cell culture of 3T3 mouse embryonic fibroblast cells (3T3 cells). (**B**) Part of the cell microarray (40 × magnification). (**C**) 120 such images (200 × magnification) were captured using auto-focusing.

To illustrate the defocusing problem in practice, we prepared a high-throughput cell microarray on polydimethylsiloxane (PDMS) via the approach shown in Fig. 1A. We used the routine two-step automatic focusing to acquire cell images at each spot. In total, 120 images were captured and 8 of them were found to be defocused at different extents. Empirically, more of these defocused images could be acquired with smaller cell densities, higher background noises of substate materials, and faster speed of autofocusing. Throughout the rest of the article, we will show how the deep-learning-based workflow of sorting and reconstruction of defocused images is established and the performance of the workflow on data collected in this section.

### 2.2 The intelligent workflow and dataset preparation

**Fig. 2:**
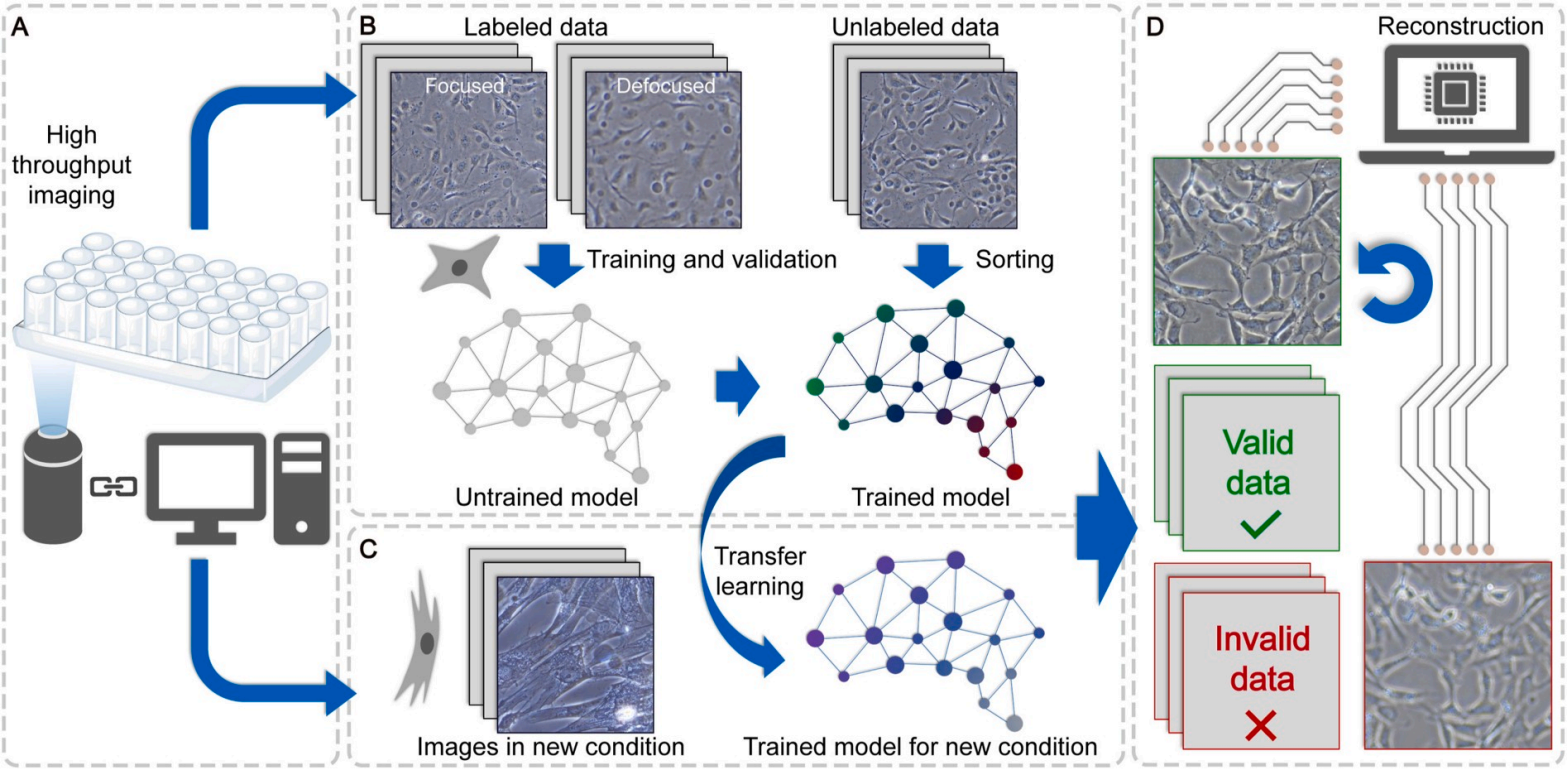
Overview of the intelligent workflow. (**A**) Images in our study were collected using an automatic high-throughput microscope. (**B**) Part of the images was used to train and validate the sorting model which was then applied to the testing set for the evaluation of classification performance. (**C**) The trained model can be fast applied to a new dataset through transfer learning requiring only a small amount of new data. (**D**) All sorted defocused images are finally applied with automatic reconstruction with another CNN model.

The intelligent workflow was presented in Fig. 2. It started with high-throughput imaging in our study but was also applicable to other experiments such as time-lapse imaging. Once the CNN models were trained on the existing data, the workflow can be used to sort and reconstruct new images produced in routine experiments. Furthermore, with a small amount of new data, models could be retrained to fast adapt to images in various conditions. To prove the practicability of our method in a variety of situations, we collected focused and defocused cell images containing 18 different conditions, including two magnifications of 100 × (10 × objective & 10 × eyepiece) and 200 × (20 × objective & 10 × eyepiece), three cell types of 3T3 cells, smooth muscle cells (SMCs), and endothelial cells (ECs) and three substrates of glass, tissue culture polystyrene (TCPS), and PDMS. Images of these conditions all showed very different characteristics caused by factors including the objective lens, cell morphologies, cell densities, and substrate textures (Fig. S1), possessing sufficient complexity to represent datasets produced in common research of biomaterials. More details about the dataset (e.g., the total numbers and ratios of images of each condition) are presented in section 4.3.

### 2.3 Sorting of defocused images with different CNN architectures and image resolutions

**Fig. 3:**
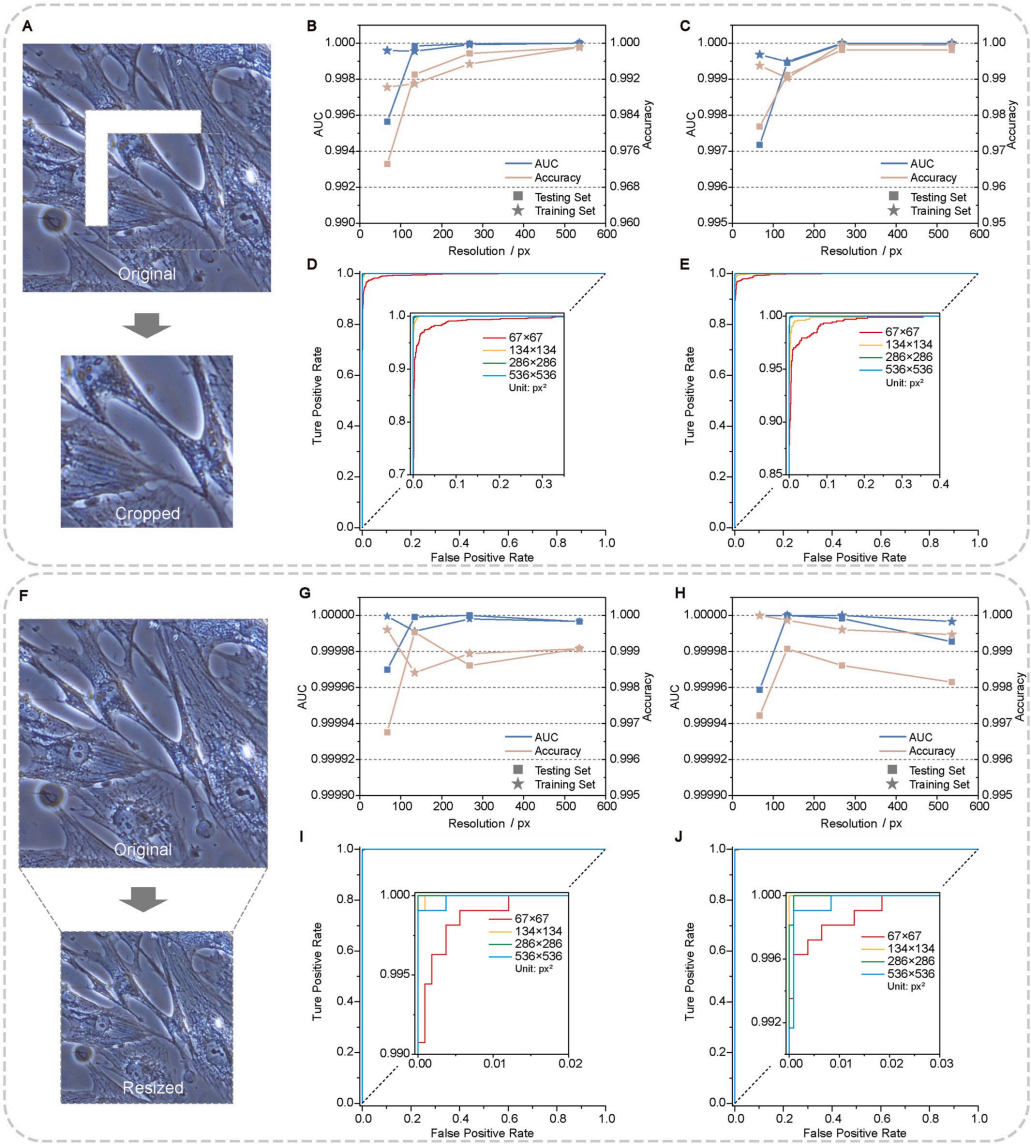
Different approaches for resolution adjustment and corresponding model performance. Results on training and testing sets are both included in figure. (**A**) Smaller images (67 × 67 px^2^, 134 × 134 px^2^, 268 × 268 px^2^) were cut from the centers of original images (536 × 536 px^2^) before input. (**B, C**) AUC and accuracy as a function of the resolution (pixel number of a single edge) of input images through cropping of (**B**) SDCNN and (**C**) ResNet50V2. (**D, E**) The ROC curves of different input resolutions through cropping of (**D**) SDCNN and (**E**) ResNet50V2. The inserts were the enlarged part of the left-upper corner of the original curves. (**F**) Original images were resized to different resolutions (the same as the cropping approach) before input. (**G, H**) AUC and accuracy as a function of the resolution of input images through resizing of (**G**) SDCNN and (**H**) ResNet50V2. (**I, J**) The ROC curves of different input resolutions through resizing of (**I**) SDCNN and (**J**) ResNet50V2. The inserts were the enlarged part of the left-upper corner of the original curves.

We assumed that classification, as a relatively mature task of deep learning, does not require a very deep and complex CNN architecture on a laboratory-scale dataset. Thus, we first used a simple SDCNN (Fig. S2) and a ResNet50V2 to compare the effectiveness of these two architectures in terms of classifying defocused cell images on the dataset of EC (the total number of training images was shown in Table S1). In addition, less training time brought higher practicability of the model, so we tried to compress the resolution of input images for the decrease of training time and to maintain the accuracy at the same time. To preserve more details, we initially cropped smaller images from the center of the original images (Fig. 3A). With the decrease of image size, the classification accuracy decreased obviously on both two models (Fig. 3B and C). The trend was also illustrated in the receiver operating characteristic (ROC) curves (Fig. 3D and E). We reckoned this was due to the reduction of image areas containing effective information, so we tried the resizing approach to adjust image resolutions for retaining holistic information as much as possible (Fig. 3F). Bilinear interpolation was used to resize images and no obvious loss of accuracy or area under the curve (AUC) was observed for both two models until resizing the resolution to 67 × 67 px^2^ (Fig. 3G and H). 134 × 134 px^2^ was chosen for the following experiments, at which resolution, the model took only approximately 1/4 time for training compared with the original resolution of 536 × 536 px^2^ (Fig. S3). Detailly, a training of SDCNN performed on a graphics processing unit (GPU) of RTX 2080 Ti required approximately 0.3 h for 50 epochs on the training set containing 7560 images (134 × 134 px^2^). We finally compared the robustness of the two models with the variation of training set size (Fig. S4). SDCNN outperformed ResNet50V2 with obvious superiority. SDCNN still maintained AUC over 0.9988 when the training set was reduced to 1/16 of the original data amount while the AUC of ResNet50V2 decreased to less than 0.99. Accordingly, SDCNN was used for further tests.

### 2.4 Transfer learning of the SDCNN model

**Fig. 4:**
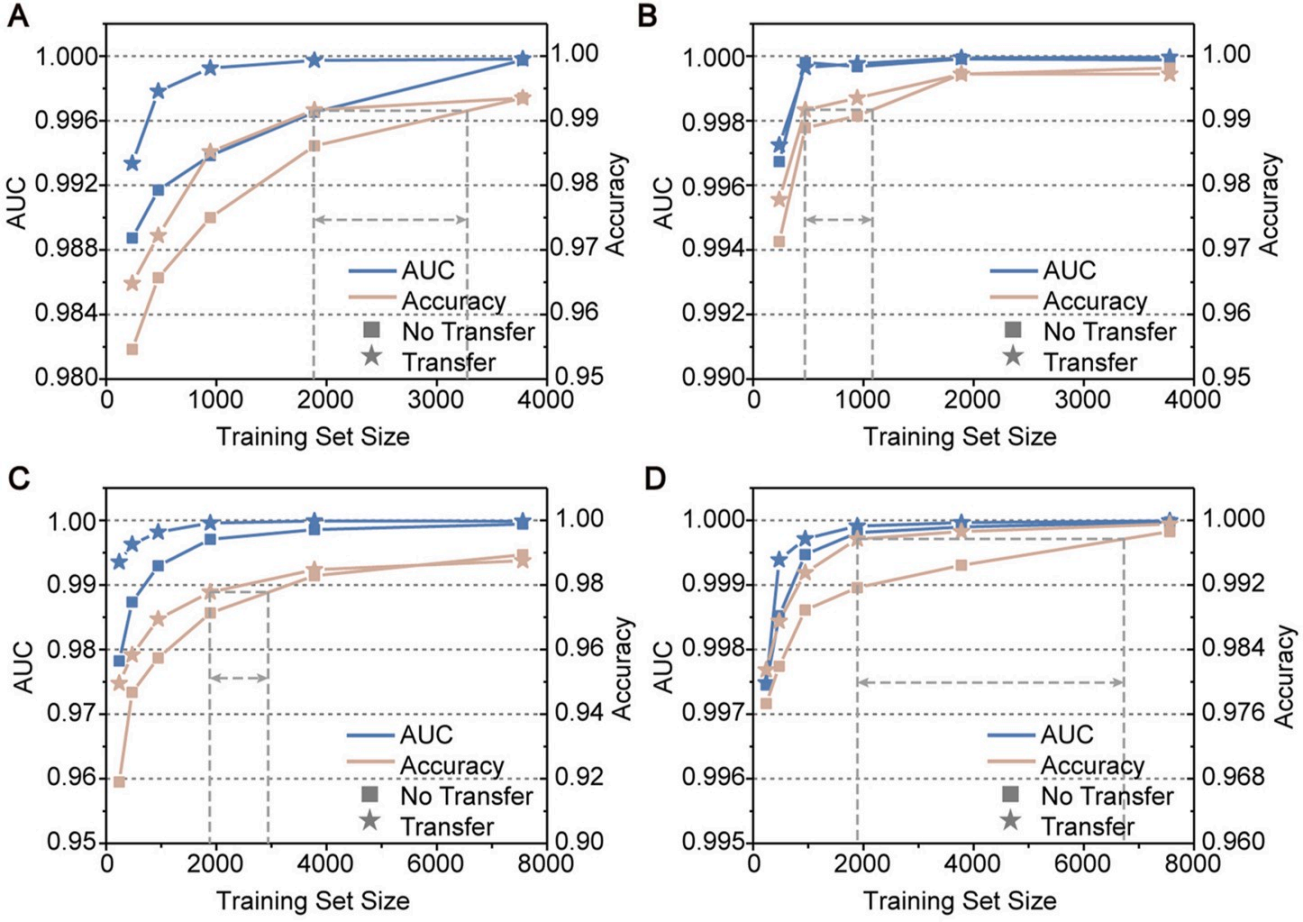
Accuracy and AUC as a function of the training set size (number of training images) through transfer learning or new training. Results on testing sets are included in figure. (**A, B**) The model was first trained on the EC dataset and then transferred to (**A**) 3T3 or (**B**) SMC dataset. (**C**) Transfer learning of models from images of 100 × magnification to 200 × magnification. Datasets of each magnification contained images of all three cells and three substrates. (**D**) Transfer learning of models from images of 200 × magnification to 100 × magnification. “No transfer” represented that the training was started from a randomly initialized model on the target dataset. The gray dotted lines with arrows illustrated the approximate data amount saved through transfer learning to reach the same accuracy.

To illustrate the practicability of rapidly applying our model to new images, we compared the data amount needed respectively for transfer learning and new training (start with a randomly initialized model) to achieve convergence. The SDCNN model trained in the last section on the EC dataset was transferred to the 3T3 or SMC dataset here (Fig. 4A and B). Compared with new training, transfer learning could save hundreds of images needed to achieve the same accuracy and AUC. The superiority of transfer learning vanishes gradually when the size of the training set reaches a certain extent. We also trained our model with images of one magnification containing all cells and substrates and then transferred the model to the dataset of another magnification (Fig. 4C and D). A similar trend was observed that transfer learning outperformed new training when the training set size was relatively small. Especially for transfer learning from 200 × magnification to 100 × magnification, near 5000 images were saved through the transfer approach (Fig. 4D). Thus, transfer learning is worth trying when the data amount is relatively limited.

### 2.5 Five-fold cross-validation on the mixed dataset

**Fig. 5:**
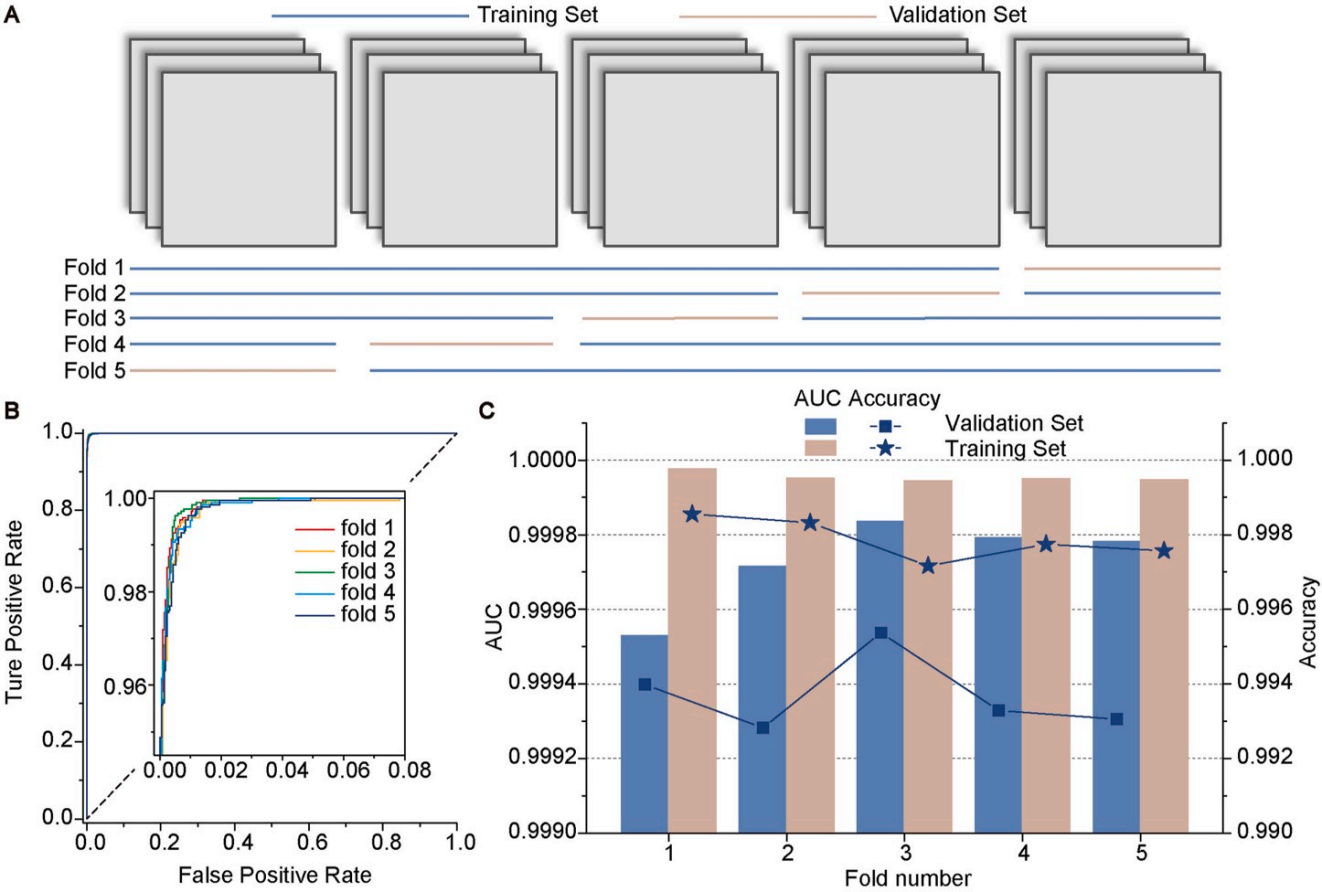
Results of five-fold cross-validation. (**A**) The dataset was randomly divided into five equal parts and one of them was set as the validation set for evaluation and the rest as the training set in turn in each fold. (**B**) The ROC curves of the five folds on validation sets. The insert was the enlarged part of the left-upper corner of the original curves. (**C**) The accuracy and AUC of different folds on training and validation sets.

High-throughput research normally covers a variety of materials or cell phenotypes, leading to images with various features within one experiment. It is not feasible that users prepare many different models for images with different features. To thoroughly evaluate the practical performance of SDCNN, we mixed up all images of the 18 different conditions and applied five-fold cross-validation on the mixed dataset (21,600 images). 20% of the dataset (4320 images) was set as the validation set in turn (Fig. 5A), and the accuracy of the model was quite robust on each fold with differences less than 0.003 (less than 0.0005 for AUC, Fig. 5B and C). Compared with training separately on datasets of different cell types (Figs. 3G and 4A, B), the AUC and accuracy had only a very moderate decrease. We randomly extracted some wrongly classified images (Fig. S5) and found that some of these images were inherently hard to define whether they were defocused or not with human eyes. Moreover, some images were labeled with wrong tags, so the accuracy should be slightly higher than presented.

### 2.6 Reconstruction of 3T3 cell images

**Fig. 6:**
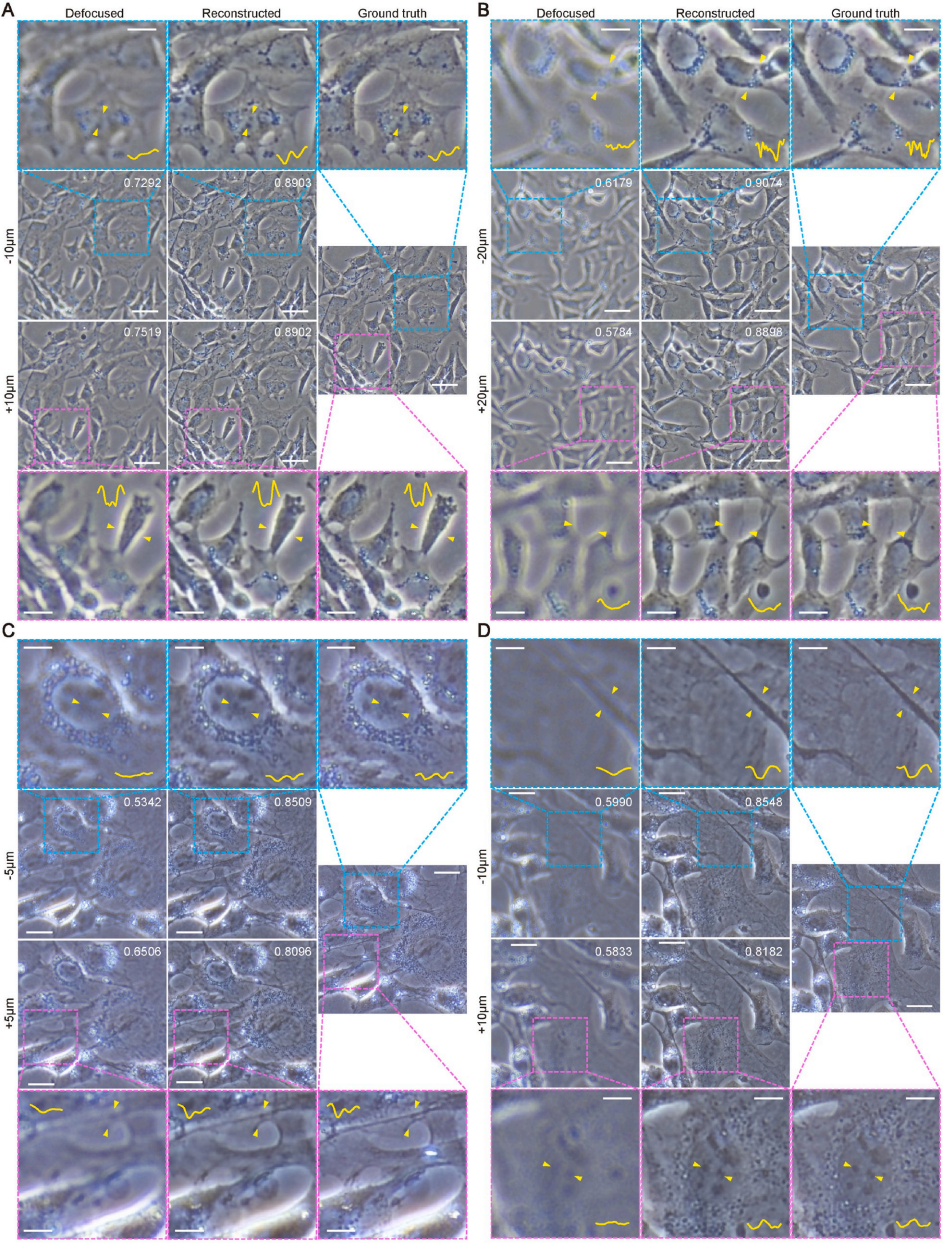
**Reconstruction performance of the ResUNet model.** (**A, B**) Reconstruction results with defocus distances of (**A**) ±10 & (**B**) ±20 μm under 100 × magnification. Scale bars are 50 μm for the original images and 20 μm for the enlarged images. (**C, D**) Reconstruction results with defocus distances of (**C**) ±5 & (**D**) ±10 μm under 200 × magnification. The inserted yellow curves were the grayscale intensity plot between two yellow arrows. The insert numbers represent the SSIM values (ranging from −1 to 1, higher is better) of the defocused and reconstructed images compared with the corresponding focused images. Scale bars are 25 μm for the original images and 10 μm for the enlarged images.

**Fig. 7:**
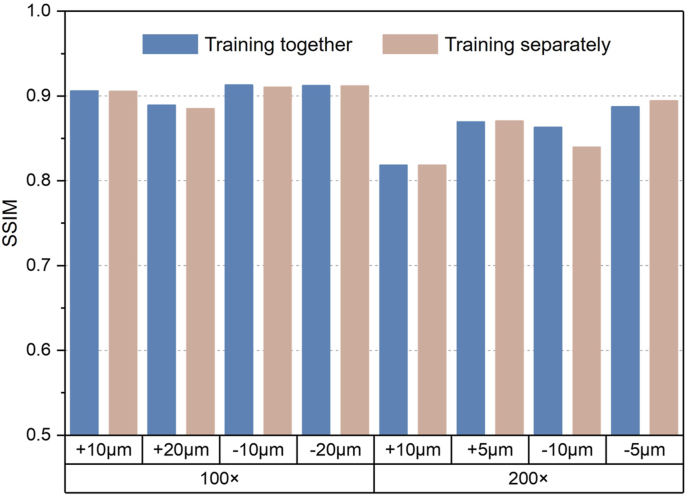
SSIM on testing sets with different training approaches. Training together represents that all images with the same magnifications were mixed together to train one model. Two models were trained and evaluated in total. Training separately represents that one model was trained for each image dataset (e.g., images of +10 μm and 100 × magnification). Eight models were trained and evaluated in total.

To achieve the image-to-image task of reconstruction of defocused images, a modified UNet architecture was used (ResUNet, Fig. S6). We introduced the improved residual structure in ResNetV2 into the original UNet architecture and added instance normalization [35] layers to normalize each input image independently. Images used to train the reconstruction model were chosen as 3T3 cells grown on the TCPS substrate. We collected focused images as training labels and defocused images taken from above and below the cell plane at fixed distances (defocus distance) as inputs. The defocus distances were determined as ±10 & ±20 μm under the 100 × magnification and ±5 & ±10 μm under the 200 × magnification and controlled by the mechanical stage automatically (±10 here represented two values of +10 & −10 instead of all values within the range. ±20 & ±5 were the same). Images with the same magnification were put together for training and the model performance was presented in Fig. 6, Fig. 7. According to the work of Zhao et al. [36], we compared L1 loss, structural similarity [37] (SSIM) loss, and a combinatory loss of L1 and SSIM for better reconstruction quality (Fig. S7). Based on the results, SSIM loss was used as the loss function of the model, and SSIM was used as the evaluation index.

After reconstruction, the SSIM of each image was significantly improved (Figs. 6 and 7, S7). From the perspective of human vision, almost all subcellular structures were reconstructed to a level that was very close to the focused images. Details including cell edges, nuclei, and textures were very clear after reconstruction while they were almost unobservable in original defocused images of ±20 μm under 100 × magnification and ±10 μm under 200 × magnification. This can also be observed in the intensity profiles of the original and reconstructed images. Even cell edges completely lost in the defocused image can still be partly reconstructed (Fig. 6B). Still, not all lost details can be restored. Small differences between reconstructed images and ground truths can be found in all images especially at places where brightness varied significantly within small areas. Notably, although we captured defocused images from four fixed defocus distances, images used in our study have a continuous variation in defocus distances due to the mechanical precision (Fig. S8). Accordingly, the model can process images of random defocus distances which accord with the real situation.

With the increase of magnification and defocus distance, the performance of the ResUNet model decreased accordingly (Fig. 7), which was reasonable because there were more details to reconstruct at higher magnification and less useful information at farther defocus distance in defocused images. We also trained the model separately with images of each defocus distance and magnification to observe whether the model performed better on the dataset with lower complexity. It was illustrated that the complexity of the dataset had no obvious influence on the model performance in our study (Fig. 7), which was an advantage of our method in practice that images generated in varied experiments could be handled with only one well-trained model in the workflow. The performance of the original UNet architecture with instance normalization layer was also tested and our model outperformed UNet in terms of SSIM for over 0.2 (Fig. S9). We also tried the Richardson–Lucy (RL) deconvolution [38] which is a classical method for image deblurring and is still used to be compared with newly developed deep-learning methods for cellular image processing [26,27]. It had poor performance on our data that the reconstructed images could not be used in any form of analysis (Fig. S10), proving the necessity of the deep-learning-based method.

To further evaluate the reconstruction ability of the ResUNet model, the reconstructed images and the ground truth images were mixed up to cheat the sorting model trained in the five-fold cross-validation (Fig. S11). When using the original focused and defocused images, the sorting model achieved the AUC of 0.9953. After replacing the defocused images with the corresponding reconstructed images outputted by the ResUNet model, the AUC of the sorting model decreased to only 0.6951, meaning that plenty of the reconstructed images were authentic enough to cheat the computer, which again illustrated the great reconstruction ability of the model. More of the reconstructed images with different features (cell densities, shapes, brightness) used to cheat the sorting model are presented in Fig. S12. We also tested the model with stitched images because whole-slide scans are also very common in high-throughput experiments. The result indicated that the splice would not cause any obvious abnormality (Fig. S13).

### 2.7 The generalization ability of the reconstruction model

**Fig. 8:**
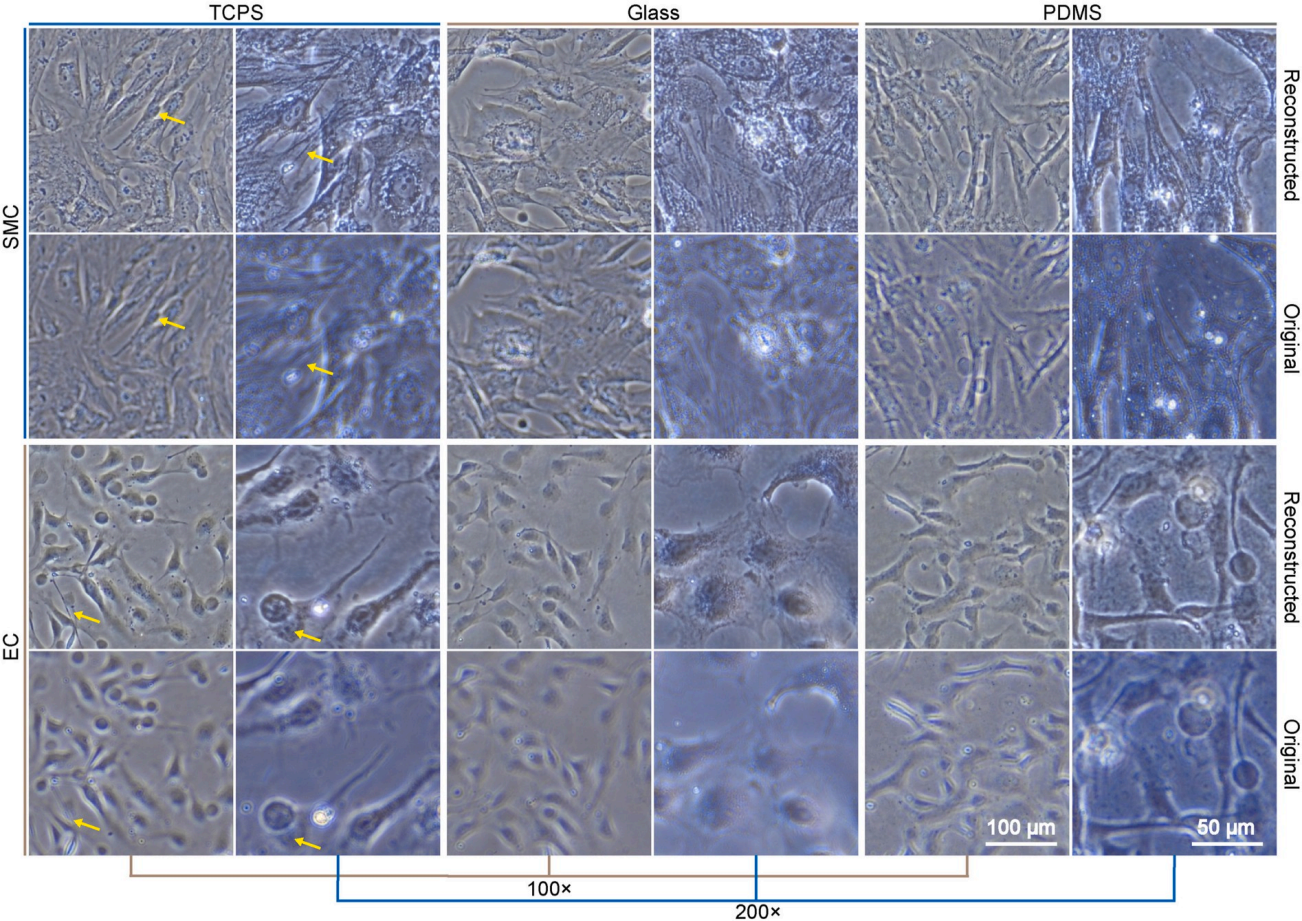
**Reconstruction performance of the ResUNet model on EC and SMC images.** The model was trained with 3T3 cell images collected specifically for reconstruction experiments on TCPS and accepted no further training before being applied to EC and SMC images. Scale bars apply to all images with the same magnification.

We directly applied the ResUNet model to EC and SMC images which were specifically collected for sorting experiments to test its generalization ability (Fig. 8). Taken overall, the model could generate images with useable quality. As pointed by the yellow arrows, cytoskeleton and cell nuclei were clearly visible, and very slim structures almost lost in the defocused images could be reconstructed as well. It was also noticed from the image of EC on PDMS (200 × ) that not in all conditions could the model be directly applied to images with new features.

### 2.8 The performance of the workflow on the high-throughput cell microarray

**Fig. 9:**
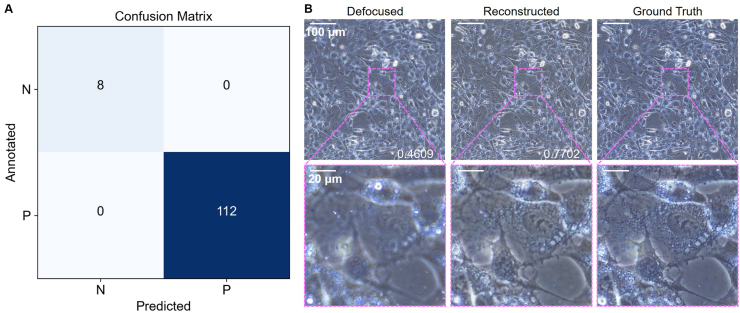
**The performance of the workflow in the high-throughput experiment.** (**A**) The confusion matrix of the sorting model. N refers to negative samples (defocused) and P refers to positive samples (focused). (**B**) The most blurry one in the 8 defocused images, the corresponding reconstructed image, and the ground truth image captured manually. The insert numbers are the SSIM values of the labeled images compared with the ground truth image.

As mentioned in section 2.1, 120 cell images were collected from different spots using automatic focusing, and 8 of them were found to be defocused. To illustrate the applicability of the workflow in practice, the models trained earlier were directly applied here. First, the sorting model trained in the five-fold cross-validation was used to sort the 120 images, and all the 8 images were successfully found and no focused images were wrongly classified as invalid data (Fig. 9A). Then, all the defocused images were reconstructed using the model trained in section 2.6, and the most defocused image was presented in Fig. 9B (The rest 7 were presented in Fig. S14). The model successfully reconstructed the image that cell nuclei and edges were very clear although the model was trained on images collected on TCPS instead of PDMS. Notably, the automatic sorting of 120 images and the reconstruction of 8 images took less than half a minute in total, which was much faster than manual sorting and re-taking. These results proved the applicability of our method in actual high-throughput experiments.

## 3 Discussion and conclusion

Here, a deep-learning-based workflow for the sorting and reconstruction of defocused cell images is presented. It has widespread usability in various experiments concerning cell imaging under phase-contrast channel that the processing of invalid data is completely automatic, requiring not a priori knowledge of imaging or optics. The sorting step in our study is to determine defocused images, while more types of invalid data can be included such as the artifacts caused by impurities or floating cells and the images containing no target cells, in which the first type of invalidity may also be reconstructed using the method. We have proved that for a regular laboratory-scale dataset, the SDCNN model is efficient enough and is also very convenient for usage that the training on a dataset containing 10,000 images takes only about 0.5 h (when resizing images to 134 × 134 px^2^). The time is even shorter for transfer learning with fewer images. When coming into a more complicated situation, our method is also flexible enough that the SDCNN can be replaced by other advanced CNN architectures to adapt to datasets containing abundant types of images which may be more and more common in the future.

Before us, researchers have addressed some of the problems related to focusing quality or super-resolution in biological images using deep learning. Yang et al. [39] evaluated the focusing quality of each object independently in fluorescent images. Regretfully, they did not offer instant solutions to defocused objects. Ozcan's group successively applied deep learning to the reconstruction of holographic images [40] and the super-resolution of fluorescent images [27]. Their work provided inspiring insights into the deep-learning-based enhancement of biological images, but cannot be directly applied to phase-contrast cell images. Zhang et al. [41] achieved a similar target of the deblurring of defocused cell images like us. However, they concentrated on small-size grayscale images of single cells collected in flow cytometry. Our model, in comparison, can achieve the one-step reconstruction of three-channel colorful images containing multiple cells with different defocusing distances. Most importantly, none of the existing studies took background noises from substrate materials, which are extremely common in biomaterials experiments and are one of the main reasons for defocusing, into account. As we achieve the subcellular-level reconstruction of phase-contrast images with an easy-to-train CNN model (single model, single loss function, and optimizer with only default parameters), the deep-learning-based ways of dealing with low-quality or invalid images are further broadened. Since three-channel color images were directly set as the output of the ResUNet model, with the complexity of the phase-contrast images in our study, it is reasonable to assume that the workflow can also be used to process other types of images in life science such as multichannel fluorescent images and stained tissue images.

The virtual reconstructed images in our study already exhibit high authenticity in terms of cheating the sorting model and human observation. If even higher authenticity of images is required, the ResUNet model may also be integrated into generative adversarial networks (GANs) as a generator like the existing studies of virtual image generation [27,30,31,42]. However, extensive discussions on the internet about a recent study of the up-sampling of photos of human faces [43] strongly suggest that GAN might fill images with details that are inherently not there. Meanwhile, GAN usually meets more difficulties in training (e.g., proper choices of more hyperparameters and loss functions) and requires more computational resources compared with a single generator model, so the use of GAN should be circumspect.

It is worth noting that there is yet no universal standard of SSIM above which the reconstructed image can be considered as completely reliable. No matter how authentic the reconstructed images are, at the current stage, we do not recommend the use of them in some biological analyses concerning calculations at pixel-level precision (e.g., the quantitative analysis of cytoskeleton textures). However, it is appropriate to use reconstructed images in the calculations of cell coverage, nucleus area, cell shape, etc. One can also use reconstructed images for further deep-learning processing such as the virtual staining or label-free classification of cells.

The development of technology brings the automation level of cell experiments to an unprecedented stage. We believe it is necessary to decrease the weight of human interventions in experimental operations to a certain degree for the increase of efficiency, precision, and repeatability. The methodology presented above is such an ideal automatic tool to deal with invalid image data generated in high-throughput experiments, that it reduces manual operations to a maximum extent. For scientists possessing no deep learning experience, the workflow can be packaged as a user-friendly program with a graphical interface, and what users need to do is putting images into the program and collecting the sorted and reconstructed images. No settings are needing specific knowledge to be done. The operations of training a new model are very alike except that the input images need to be tagged by users in advance.

## 4 Materials and methods

### 4.1 Substrate preparation

Sterile tissue culture polystyrene (TCPS, 6-well plate, Cat No. 140675, ThermoFisher, U.S.A.) was directly used in cell culture. Glass slides were dipped in the Hellmanex Ⅲ solution (0.5 wt%, Hellma, Germany) at 60 °C for 30 min for clearance, and then washed carefully with deionized water repeatedly. All glass slides were then stored in ethanol. Before cell culture, all samples were dried with nitrogen flow, put into TCPS 6-well plate, and placed under an ultraviolet lamp for 30 min for sterilization. The curing agent and prepolymer of PDMS (SYLGARD 184 Silicone Elastomer Kit, Dow Corning, U.S.A.) were mixed in 6-well TCPS plates at the mass ratio of 1:10 and put into a vacuum environment to discharge air bubbles. Then the samples were solidified under 75 °C for 4 h. For cell adhesion, all PDMS samples were treated with air plasma with a power of 100 W for 30 s in a plasma cleaner (PT-5S, Sanhoptt, China). For the preparation of the high-throughput chip, the mixture of the curing agent and prepolymer of PDMS was spin-coated and solidified on a standard glass slide, and a designed metal mask was covered on it during the air plasma treatment. All PDMS samples were stored in deionized water to maintain the effect of plasma treatment. They were applied with the same sterilization operation as glass slides before cell culture. At least three wells were prepared for the imaging of each type of sample except for the high-throughput chip (one sample was prepared).

### 4.2 Cell culture

Human umbilical vein endothelial cells (ECs) and human umbilical artery smooth muscle cells (SMCs) were purchased from ScienCell Research Laboratories (U.S.A.) and both cell types used for experiments were between 3 and 5 passages. The NIH 3T3 mouse embryonic fibroblast cell line was obtained from the Chinese Academy of Sciences (China). All three cells were cultured at a density of 30,000/cm^2^. Cells were allowed to adhere for 4 h in endothelial cell medium (ECM, Cat No. 1001, ScienCell, U.S.A.) for ECs, smooth muscle cell medium (SMCM, Cat No. 1101, ScienCell, U.S.A.) for SMCs, and high-glucose DMEM medium (Cat No. CR-12800, Cienry Biotechnology, China) with 10% fetal calf serum (Cat No. 11011–8611, TIANHANG Biology, China) for 3T3 at 37 °C in a humidified atmosphere containing 5% CO2. After incubation, all samples were washed three times with PBS and fixed with 4% paraformaldehyde at 4 °C for 15 min. Again, samples were washed with PBS three times and stored in PBS. All samples were used for image acquisition within 24 h after fixing.

### 4.3 Image acquisition and preprocessing

An inverted microscope (ECLIPSE Ti2, Nikon, Japan) controlled by the NIS-Elements software (Nikon, Japan) was used to acquire phase-contrast images of cells. Objective lenses of 10 × /0.30-NA and 20 × /0.45-NA were separately used to acquire images of different magnifications. For images used for sorting, all focused and defocused images were randomly taken from substrates. To ensure the quality and balance of our dataset, all focusing processes were manual operations to obtain precisely focused images and sufficient defocused images. The defocus degree of each image was controlled by turning the focusing knob randomly. The defocus distances ranged from a few micrometers to tens of micrometers. The ratio of focused and defocused images and the ratio of images of two different magnifications were both 1:1. The ratio of images collected from the three substrates was 1:1:1. The ratio of images of three cells was 2:1:1 (EC to SMC to 3T3). For images used for reconstruction, five images were taken from each point of substrates, which were a single focused image and four defocused images taken from above and below the cell plane at different distances (±10 μm and ±20 μm under 100 × magnification). Because of operation mistake, defocused images of ±5 μm and ±10 μm under 200 × magnification were taken separately at different points, but this does not influence the following experiments. The defocus distances were chosen manually and controlled by the mechanical stage of the microscope. We chose these distances because empirically, the defocusing caused by the automatic microscope will not exceed the maximal distance used in our study. This can also be observed from Figs. 7 and 9, and Fig. S14 that no image captured from the cell microarray was more defocused than images collected for model training. We chose different defocus distances for these two magnifications because the degree of blur in the image was larger at higher magnification compared with lower magnification at the same defocus distance. For the high-throughput experiment, 120 images were collected using the 2-step fast autofocusing in the NIS-Elements software. Each of them was collected from different spots in the cell microarray. 8 in the 120 images were observed to be defocused and were annotated as negative samples manually. All images mentioned above were of the resolution of 1608 × 1608 px^2^ (595 × 595 μm^2^). For data augmentation, in sorting experiments, each image was cropped into 9 images of 536 × 536 px^2^. In reconstruction experiments, each image was cropped into 16 images of 388 × 388 px^2^. All cropped images were divided into training sets, validation sets, and testing sets randomly (Specific numbers of images in different sets were presented in Table S1). To adapt to the reconstruction model, images collected from the high-throughput chip were cropped into 1604 × 1604 px^2^.

### 4.4 Characterization and deep learning models

All training processes were performed on an RTX 2080 Ti GPU with a TensorFlow 2.0 (https://tensorflow.google.cn/) environment. The training for the sorting model took 0.3–3 h depending on the resolution of images and the network architecture used (7560 images in the training set). Training for the reconstruction model with images of each magnification (5500–6000 images in the training set) took about 22 h. The information used to draw ROC curves and the values of AUC of sorting models were obtained using the roc_curve function in the sklearn [44] library (https://scikit-learn.org/stable/). The grayscale intensity profiles in Fig. 6 were measured by the Fiji [45] opensource software (https://fiji.sc/). For the RL deconvolution, we used the richardson_lucy function in the skimage [46] library (https://scikit-image.org/). A Gaussian filter was used as the point spread function (PSF) needed in the calculation of RL deconvolution. The actual PSF of the microscope was unknown to us, so we wrote a python script for the grid searching for the best parameters of the RL deconvolution. Parameters including the size of the Gaussian filter, the standard deviation of the Gaussian filter, and the number of iterations of the richardson_lucy function were searched. We chose different parameters for each channel of each image based on the quality of generated images. All chosen parameters are listed in Table S2.

*SDCNN.* We assumed that the sorting of focused and defocused images was a relatively simple classification task that did not require a complicated design of network architecture. Accordingly, we used only basic layers including the input layer, the 2D convolutional layer, the max-pooling layer, and the fully-connected layer (Fig. S2). We also used the global average pooling layer to replace the regular flatten operation to reduce the number of trainable parameters. We applied one-hot encoded labels on our data ([1, 0] for focused images and [0, 1] for defocused images), so the final output layer had two nodes and was activated with a softmax function. Categorical cross-entropy was set as the loss function accordingly and a sgd optimizer was used to minimize it. For both SDCNN and ResNet50V2, the initial learning rate was set as 0.001 and set to decrease by half every five epochs. The momentum was set as 0.9. The batch size was set as 4 for each iteration. The model that achieved the lowest loss value on the validation set within 50 epochs was saved for the following experiments. There was no testing set in five-fold cross-validation, so models were trained on the training set for 50 epochs and then tested on the validation set. For the fine-tuning in transfer learning experiments, the initial learning rate was set as 0.0002 and decreased to 0.0001 after 25 epochs. For clarity, in the initial training of the transfer learning process, all images in one dataset (e.g., the EC dataset containing 7560 images) were used for training. Then the trained model was fine-tuned on other datasets using different ratios of the dataset (e.g., 1/2, 1/4, 1/8, and 1/16 of the SMC dataset containing 3780 images) to observe the efficiency of model transfer. Resizing and cropping of images were performed using the image.resize and the image.central_crop application program interface (API) in the TensorFlow library during the training process. Before input, image.per_image_standardization API was used to scale all data to the distribution of 0 ± 1 (mean ± S.D.).

*ResUNet.* The UNet architecture was originally designed for biomedical image segmentation but then proved useful in many other tasks concerning finding the connections between input images and output images including virtual fluorescent image generation [24,25], virtual histological staining [30,32], and resolution enhancement [27,42]. The skip connection in ResNet architecture was proved to be beneficial for the gradient descent process which can be observed from the visualized loss landscape [47]. We introduced the residual block into the UNet architecture to obtain an easy-to-train model for defocused image reconstruction. The original down-sampling process in UNet was realized through two continuous convolution operations and then a max-pooling operation as below.

$$\text{y}=\text{MaxPool}\left[\text{ReLU}\left\{{\text{Conv}}_{3\times 3}\left(\text{ReLU}\left\{{\text{Conv}}_{3\times 3}\left(\text{x}\right)\right\}\right)\right\}\right]$$

where x represents the output of the last layer and y represents the input of the next layer. we replaced the two convolution operations with a modified residual block containing two convolution operations and pre-normalization and pre-activation before convolution. Considering that the reconstruction process was an image-to-image task, we used instance normalization instead of batch normalization to maintain the style and distribution of every single image. Because the number of filters kept changing in each residual block, channels of the input of each residual block were adjusted to the same as the output by a 1 × 1 convolution operation. The whole process can be represented as below.

$${\text{y}}_{1}={\text{Conv}}_{3\times 3}\left(\text{ReLU}\{\text{InsNorm}[{\text{Conv}}_{3\times 3}\left(\text{ReLU}\left\{\text{InsNorm}\left[\text{x}\right]\right\}\right)]\right\})$$

$${\text{y}}_{2}={\text{Conv}}_{1\times 1}\left(\text{ReLU}\{\text{InsNorm}[\text{Crop}\left(\text{x}\right)]\right\})$$

$${\text{y}}_{3}=\text{MaxPool}\left[\text{y}1+\text{y}2\right]$$

where x represents the output of the last layer, y_1_ and y_2_ represent the output of two paths of a residual block, and y_3_ represents the input of the next layer. The same replacement of original convolution operations by residual blocks was applied at the up-sampling process after each transpose convolution operation. The more detailed ResUNet structure is presented in Fig. S6. For comparison of the performance of the ResUNet and the original UNet in our task, we added the instance normalization layer after the convolutional layer in the original UNet model otherwise the loss decreased little in training. From the aspect of human vision, structural similarity (SSIM) describes the similarity between images better than the mean absolute error or mean squared error. Because the optimizer aims to minimize the value of the loss function, SSIM cannot be directly used. Instead, we used SSIM loss as the optimization target, which is defined as below.

SSIM_Loss=1−SSIM(y_true, y_pred)

where y_ture represents the focused image and y_pred represents the prediction of the model. The SSIM value was calculated using the tensorflow.image.ssim API. We also tried L1 loss and a combinatory loss function containing L1 loss and SSIM loss which is defined as below.

Combinatory_Loss=L1_Loss+100×SSIM_Loss

SSIM loss was multiplied by 100 to increase its weight in the combinatory loss for balance. Nevertheless, SSIM loss achieved the highest SSIM value among all three loss functions on the testing set and even lower mean absolute error (MAE) than the combinatory loss (Fig. S7). Because the size of images kept decreasing due to convolution operations, the resolutions of input images (572 × 572 px^2^) and output images (388 × 388 px^2^) were not the same. To avoid the loss of image areas, we padded original images (388 × 388 px^2^) with the symmetric method to 572 × 572 px^2^ so that output images of the same size as original ones could be obtained. A Nesterov-accelerated adaptive moment estimation (Nadam) optimizer was used to minimize the target loss with default parameters provided in TensorFlow API. Images with the same magnification were mixed together for training and the model with the highest SSIM on the validation set within 60 epochs was saved for further tests. The batch size for each iteration was set as 2 considering memory restriction. When training separately on the dataset of each magnification and each defocus distance, the epoch number was adjusted to 40 considering that the overfitting emerged earlier on a smaller dataset.

For the demonstration of the workflow on the high-throughput cell microarray, the sorting model trained in fold-3 (Section 2.5, Fig. 5) was used because it achieved the highest accuracy on the validation set, and the model of “training together, 200 × magnification” (Section 2.6, Fig. 7) was used for reconstruction. The sorting model was trained on images of 536 × 536 px^2^. To adapt to the sorting model, for the 120 images of 1604 × 1604 px^2^, only the central part of the image (536 × 536 px^2^) was used as the inputs. After sorting, the defocused images were directly inputted into the reconstruction model without cropping or resizing.

### 4.5 Code and data availability

All images used to train our models are available at https://figshare.com/s/8633708789dcc4510c9b. Deep learning codes used in our study are available at https://github.com/XueYunfan/Intelligent-Sorting-and-Reconstruction-of-Defocused-Cell-Images.

## Declaration of interests

The authors declare that they have no known competing financial interests or personal relationships that could have appeared to influence the work reported in this paper.

## CRediT authorship contribution statement

**Yunfan Xue:** Conceptualization, Methodology, Software, Validation, Formal analysis, Investigation, Data curation, Writing – original draft, Writing – review & editing, Visualization. **Honglin Qian:** Conceptualization, Methodology, Validation, Formal analysis, Investigation, Writing – review & editing. **Xu Li:** Investigation, Writing – review & editing. **Jing Wang:** Conceptualization, Writing – review & editing. **Kefeng Ren:** Conceptualization, Supervision, Funding acquisition, Writing – review & editing. **Jian Ji:** Conceptualization, Supervision, Funding acquisition, Project administration, Writing – review & editing.

## Declaration of competing interest

The authors declare no conflict of interest.
