# Supplementary material for: A deep-learning-based workflow to deal with the defocusing problem in high-throughput experiments
Source: Bioact Mater. 2021 Sep 16;11:218–29. doi: 10.1016/j.bioactmat.2021.09.018 (PMC8665348; doi:10.1016/j.bioactmat.2021.09.018)
Supplement: Multimedia component 1 [file mmc1.docx]

Supporting Information

A deep-learning-based workflow to deal with the defocusing problem in high-throughput experiments

Yunfan Xue^1†^, Honglin Qian^1†^, Xu Li^1^, Jing Wang^1^, Kefeng Ren^1^, and Jian Ji^1^*

^1^MOE Key Laboratory of Macromolecule Synthesis and Functionalization, Department of Polymer Science and Engineering, Zhejiang University, Hangzhou, 310027, PR China.

*E-mail: [jijian@zju.edu.cn](mailto:jijian@zju.edu.cn)

^†^These two authors contributed equally.


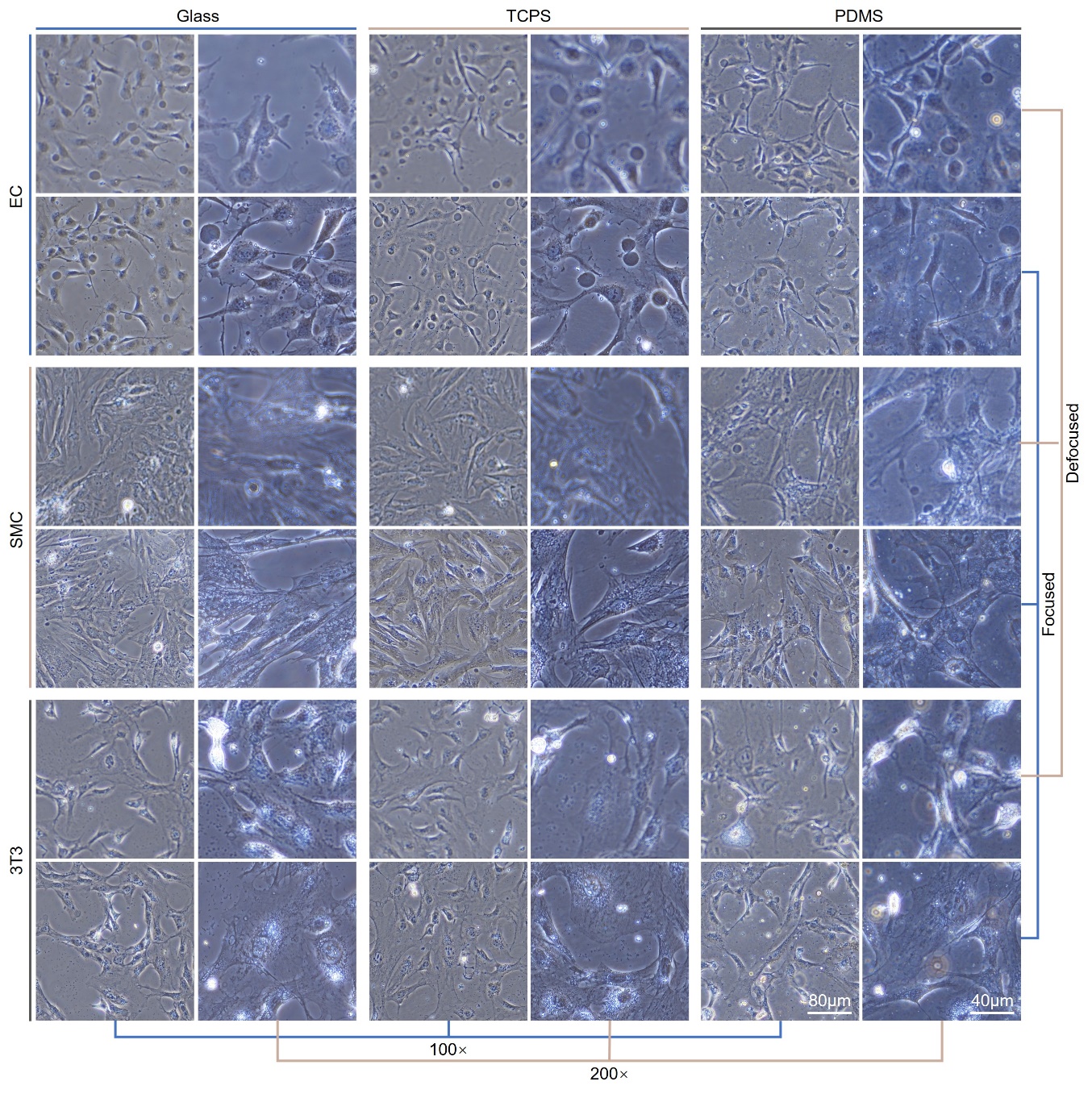


**Fig. S1.** Part of the images collected for sorting experiments.


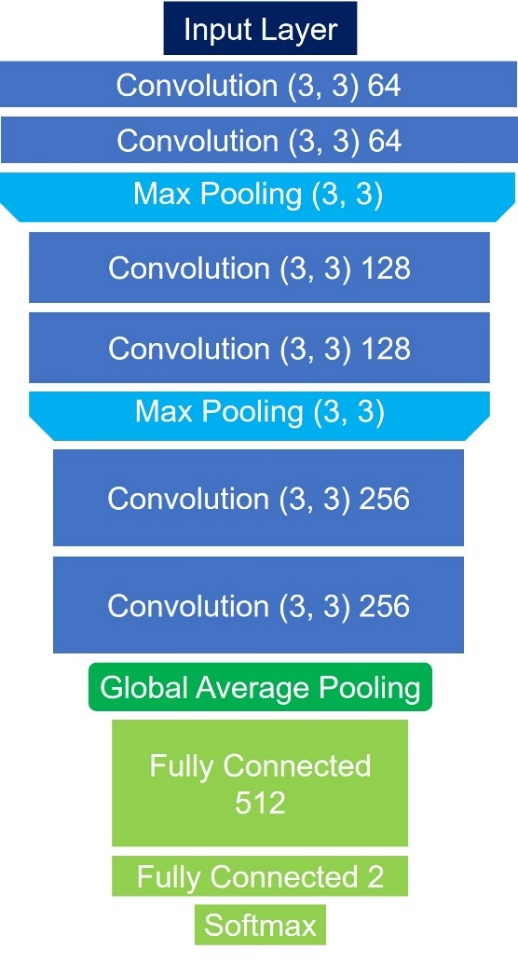


**Fig. S2.** The structure of the self-defined convolutional neural network (SDCNN).


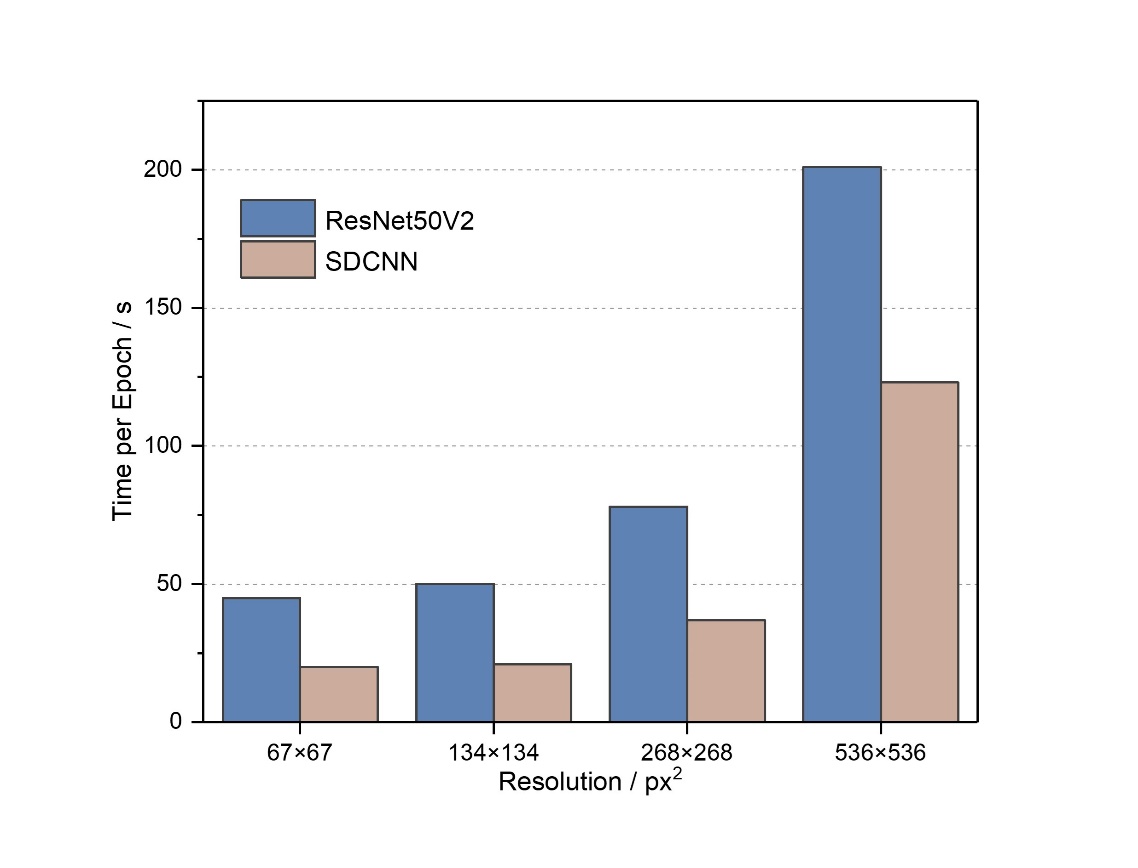


**Fig. S3.** Training time needed for each epoch on the EC dataset with different image resolutions. The batch size was set as 4 and 1890 iterations were needed for each epoch.


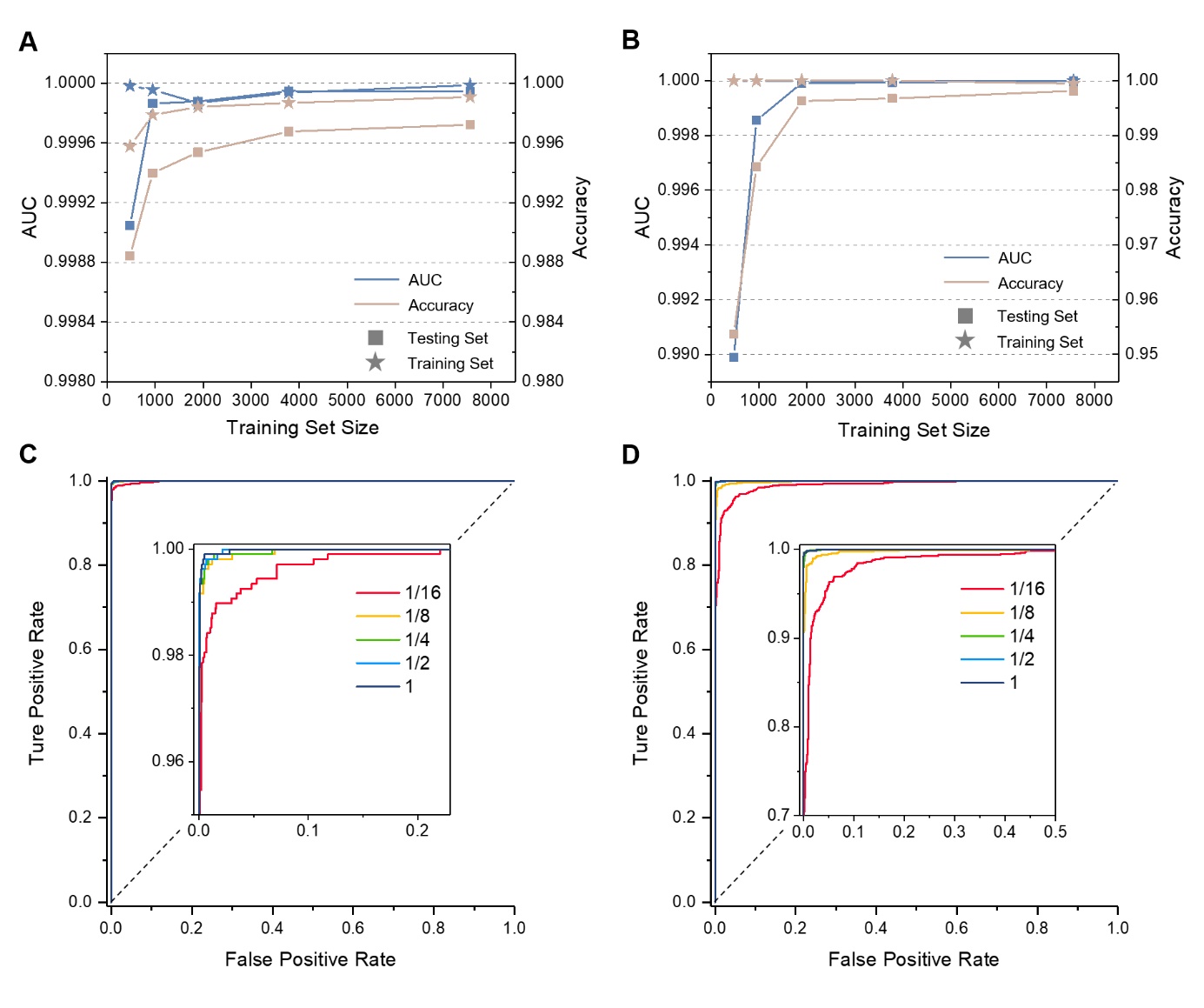


**Fig. S4.** Performance of SDCNN and ResNet50V2 on the same testing set with different training set sizes. (**A, B**) AUC and accuracy as a function of training set size (number of images) of (**A**) SDCNN and (**B**) ResNet50V2. (**C, D**) The ROC curves of different training set sizes (compared with the original training set containing 5760 images) of (**C**) SDCNN and (**D**) ResNet50V2. The inserts were the enlarged part of the left-upper corner of the original curves. All images were resized to 134×134 px^2^ before input.

**
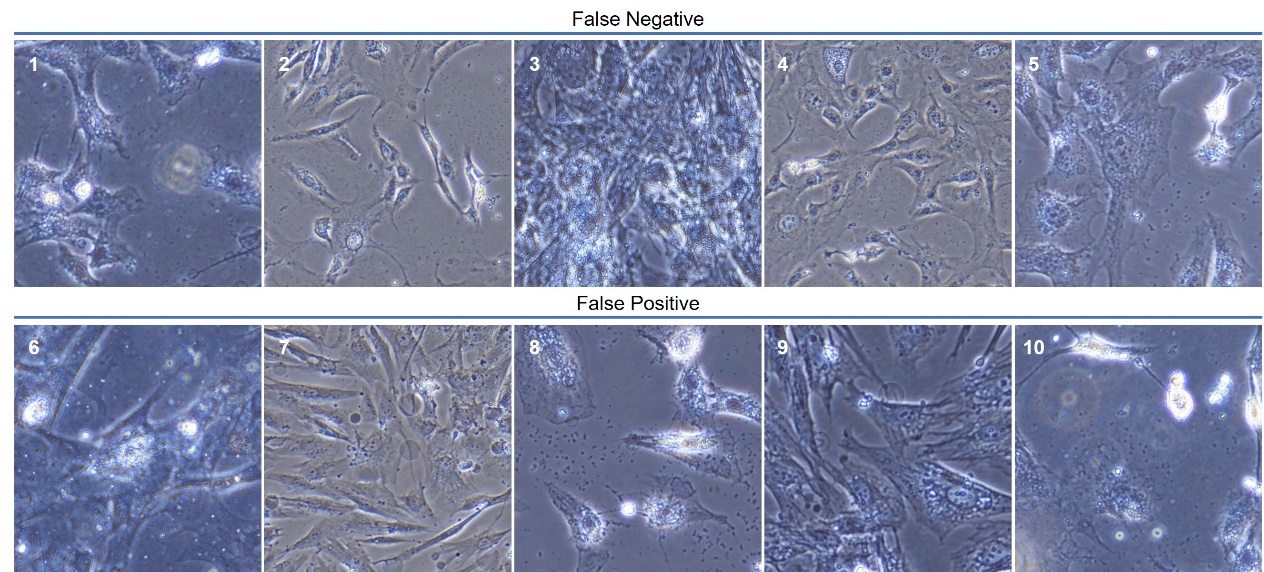
**

**Fig. S5.** Part of the wrongly classified images. Positive represents focused and negative represents defocused. Part of the images was inherently hard to determine whether they were defocused or not (2, 4, 5, 7, 8, 10). Some defocused images were tagged with the wrong label of positive (1, 3). This was caused by two reasons. Firstly, the cell plane was not completely parallel to the focal plane because of mechanical precision so that some areas in original images (1608×1608 px^2^) might be slightly defocused, leading to some defocused images (536×536 px^2^) with positive tags. Secondly, cells were overlapped when local density was very high that it was impossible to focus on all cells in images at the same time. The two reasons were also applicable to focused images with negative labels (7, 8, 10).


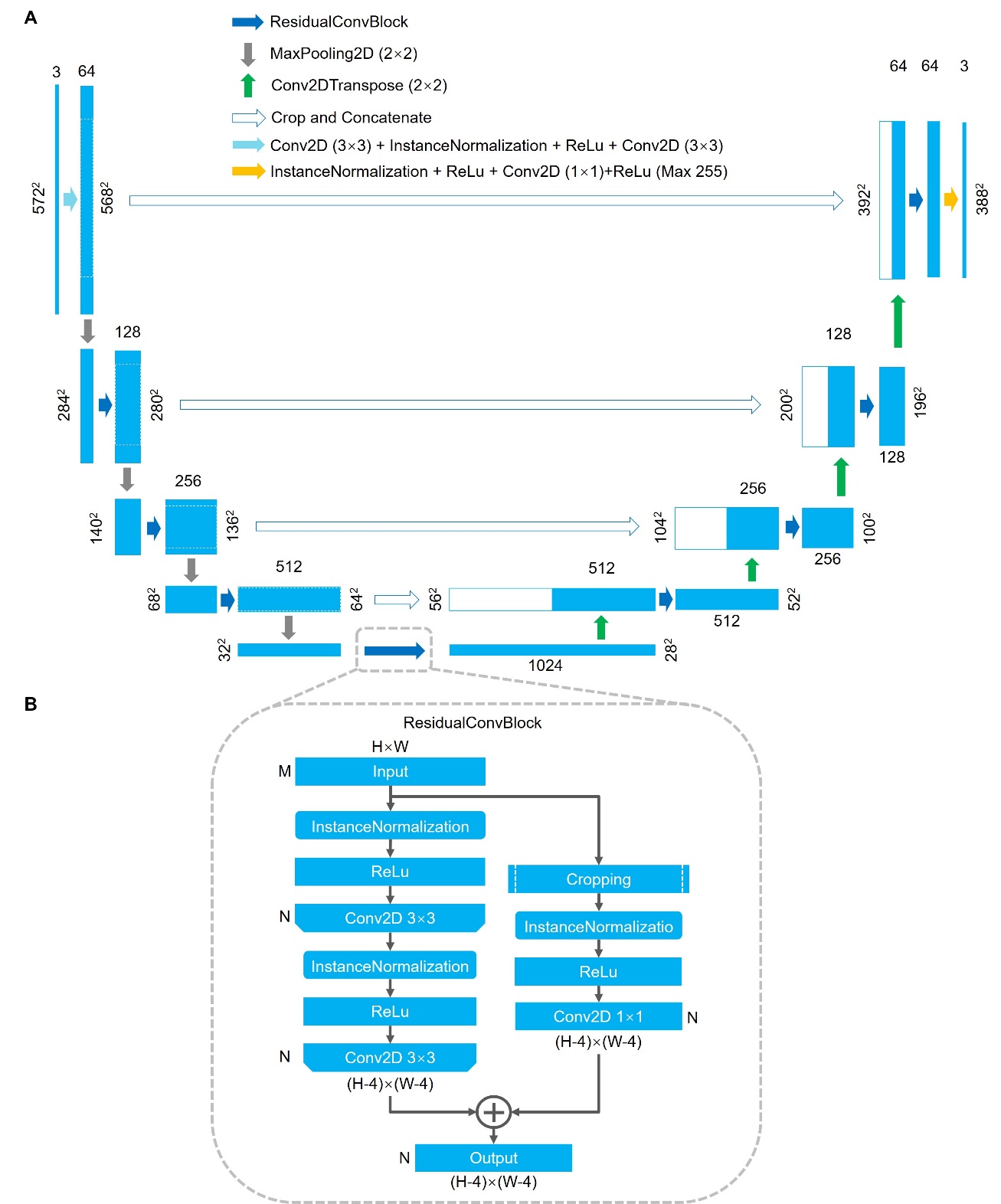


**Fig. S6.** Structure of the reconstruction model. (**A**) the ResUNet architecture and (**B**) the residual block. H and W represent the height and width of feature maps. M and N represent channel numbers.


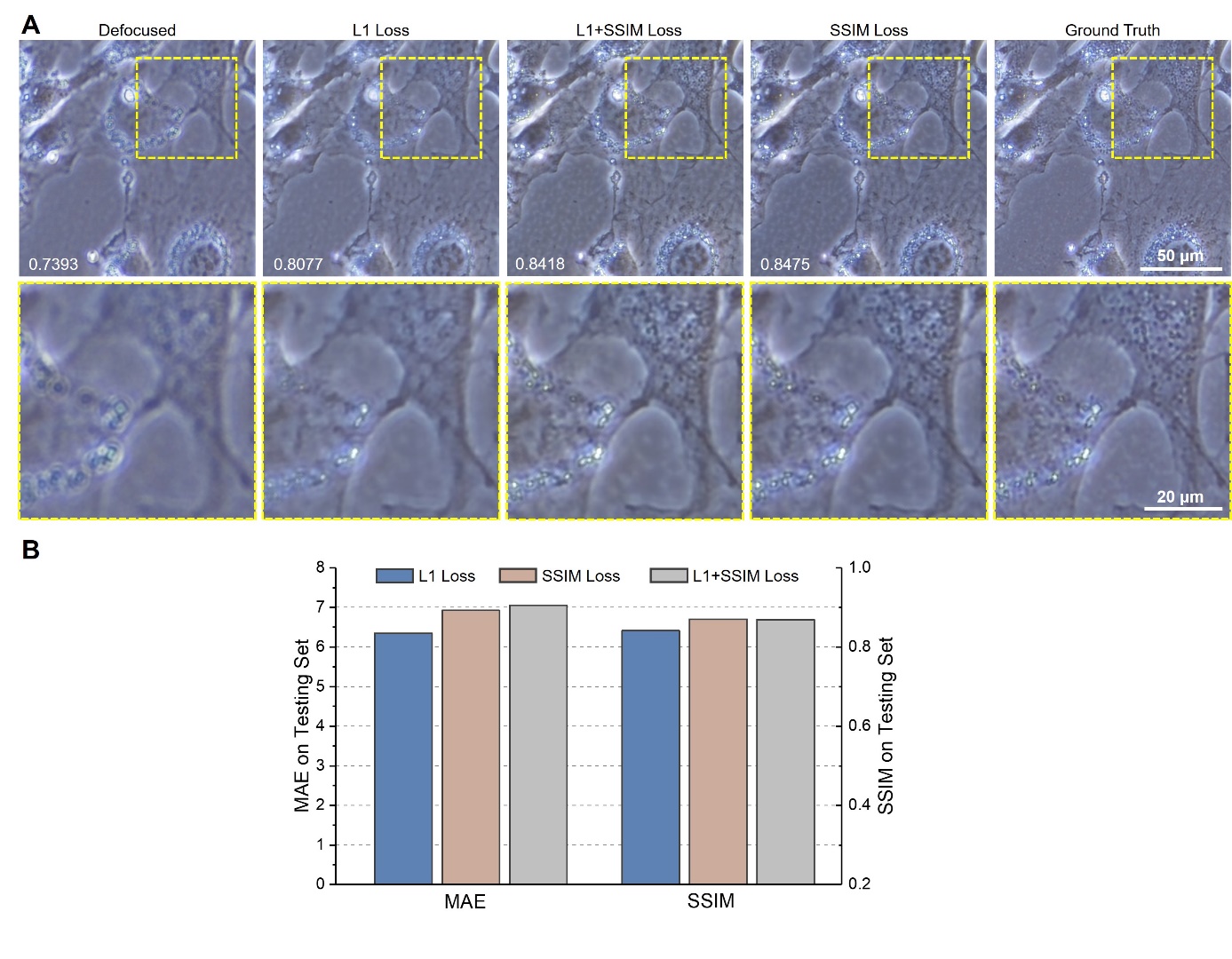


**Fig. S7.** Performance of ResUNet with different loss functions. (**A**) The reconstruction performance using different loss functions on the same image. The insert numbers are the SSIM of the tagged image compared with the ground truth (focused image). (**B**) The SSIM and mean absolute error (MAE) on the testing set (+5 μm, 200× magnification) using different loss functions. SSIM loss achieved the highest SSIM value and lower MAE value compared with the combinatory loss (L1&SSIM Loss).


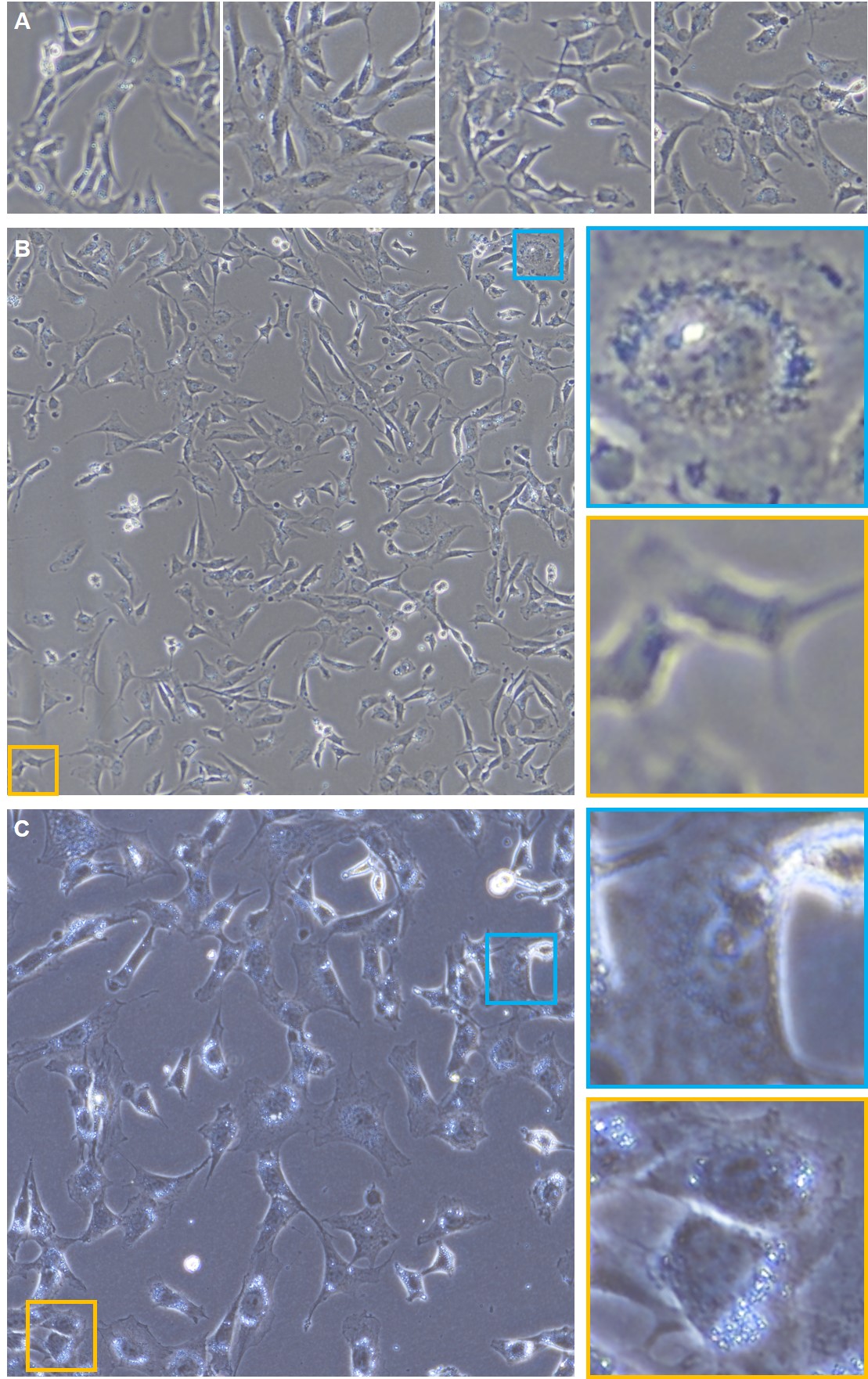


**Fig. S8.** Images used to train and test the reconstruction model have unfixed defocus distances. As mentioned in Fig.S5, this is because that the cell plane was not completely parallel to the focal plane limited by mechanical precision. (A) Some images in the dataset of +10 μm 100× magnification. They have different defocus distances although are from the same dataset. (B, C) The tilt can be observed from different areas of the uncropped original images. (B) 100× magnification and (C) 200× magnification.

**
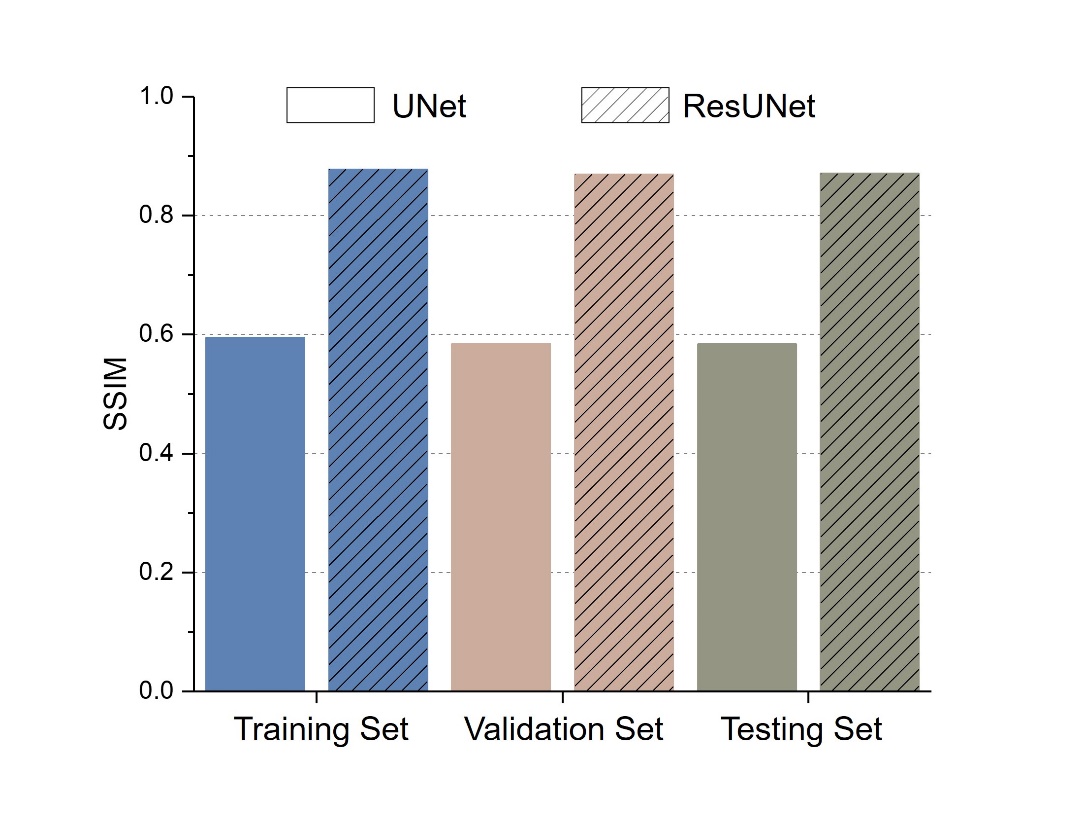
**

**Fig. S9.** SSIM of the ResUNet and UNet models on the dataset of +5 μm and 200× magnification.


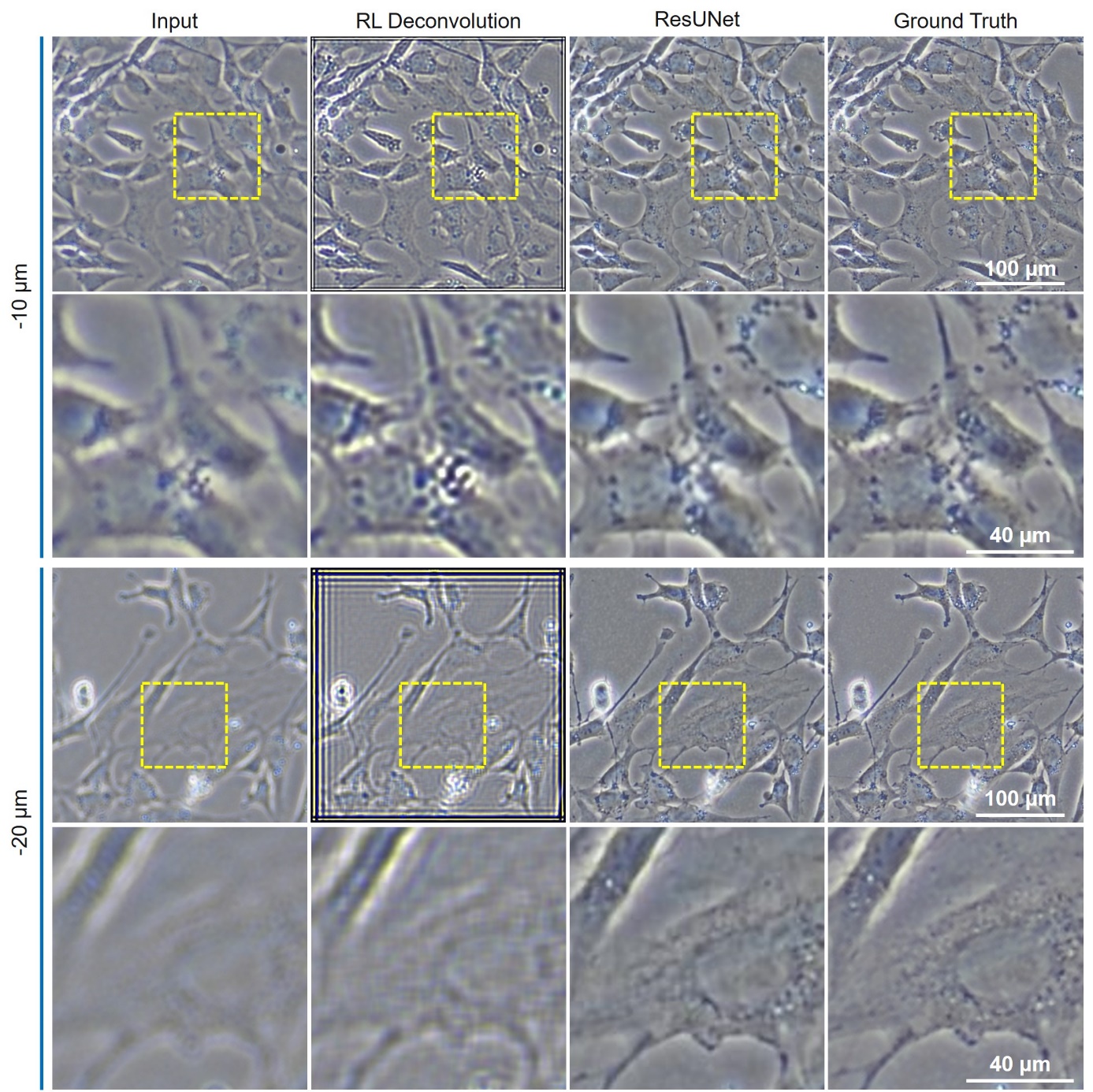


**Fig. S10.** Qualitative comparison of the reconstruction ability of the deconvolution approach and the deep learning approach. RL refers to Richardson–Lucy. Both two images used for the comparison were of 100× magnification but were of different defocus distances. A Gaussian filter was used as the point spread function (PSF) in the calculation of RL deconvolution.

**
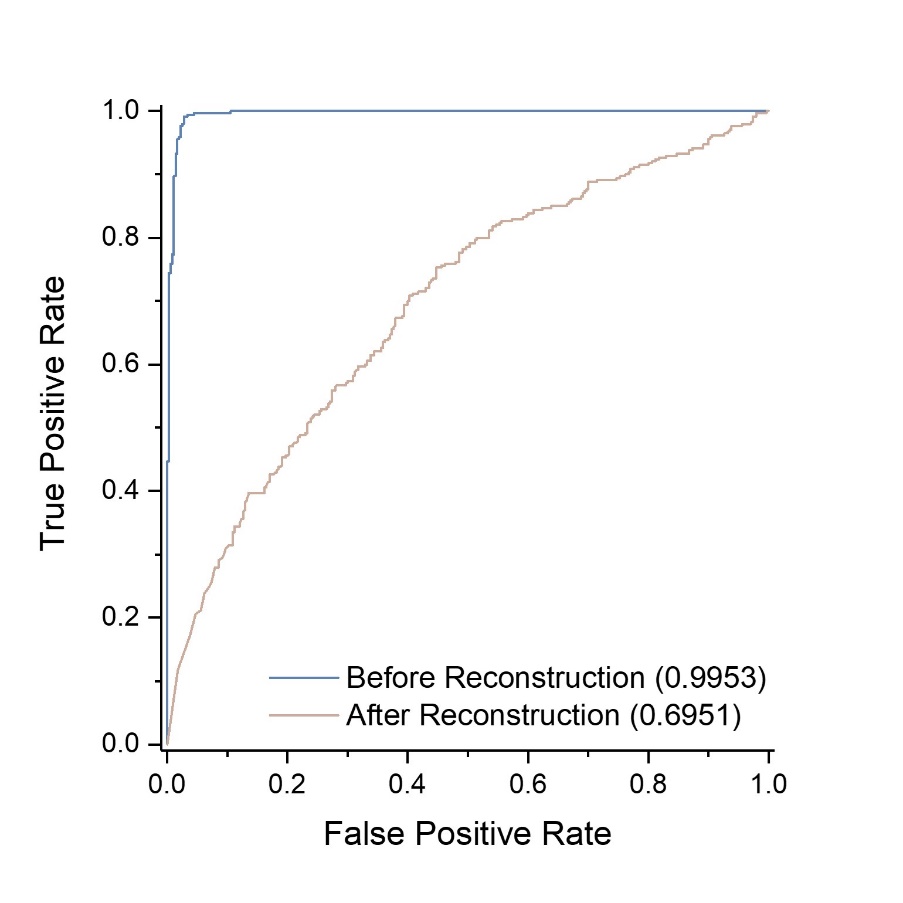
**

**Fig. S11.** ROC curves of applying the sorting model (trained in five-fold cross-validation) on focused and defocused 3T3 cell images. 340 focused images and 340 defocused images were randomly chosen from testing sets prepared for reconstruction experiments. Before reconstruction, all images were directly used for sorting to calculate AUC. After reconstruction, all defocused images were replaced with their reconstructed version and then used for sorting. The sorting model showed a significant decrease in classification performance after reconstruction (AUC in brackets), meaning that plenty of the reconstructed images were authentic enough to cheat the computer.


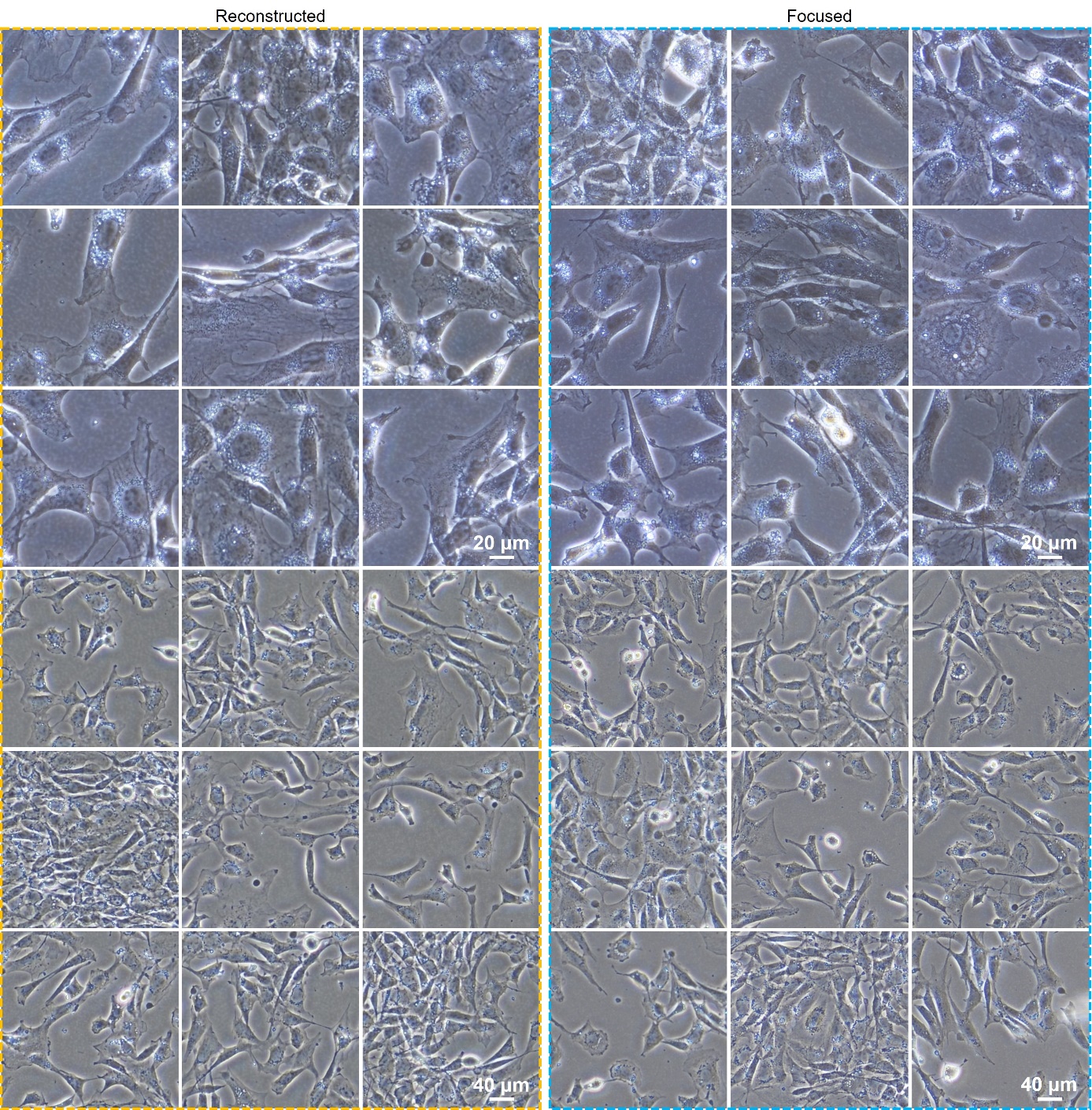


**Fig. S12.** Part of the reconstructed images and focused images used to cheat the sorting model.


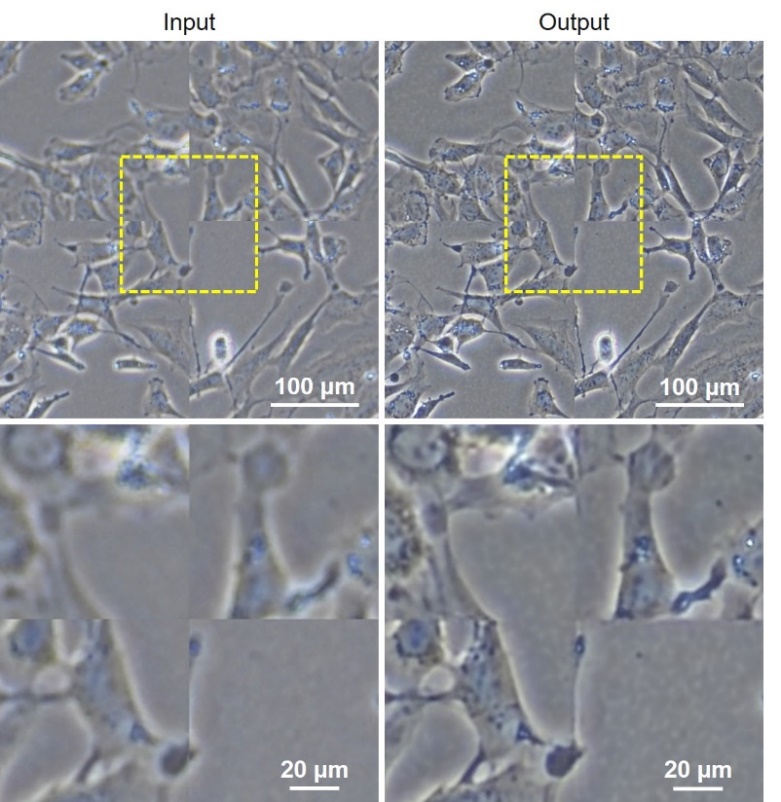


**Fig. S13.** The performance of the ResUNet on a stitched image. Whole-slide scans are common in high-throughput experiments. An extreme example was used to illustrate that the model can process stitched images. Four irrelevant images (100× magnification, -10 μm) in the testing set were stitched together and then the stitched image was directly sent into the model. The splice did not cause any obvious abnormality. However, we believe the best way to deal with stitched images is to crop them into separated images along with the splices because the stitching parameters are normally known and set by users in automatic microscopes (e.g., a large image of 4,550×4,550 px2 with 10% overlapping is stitched from 100 images of 500×500 px^2^).


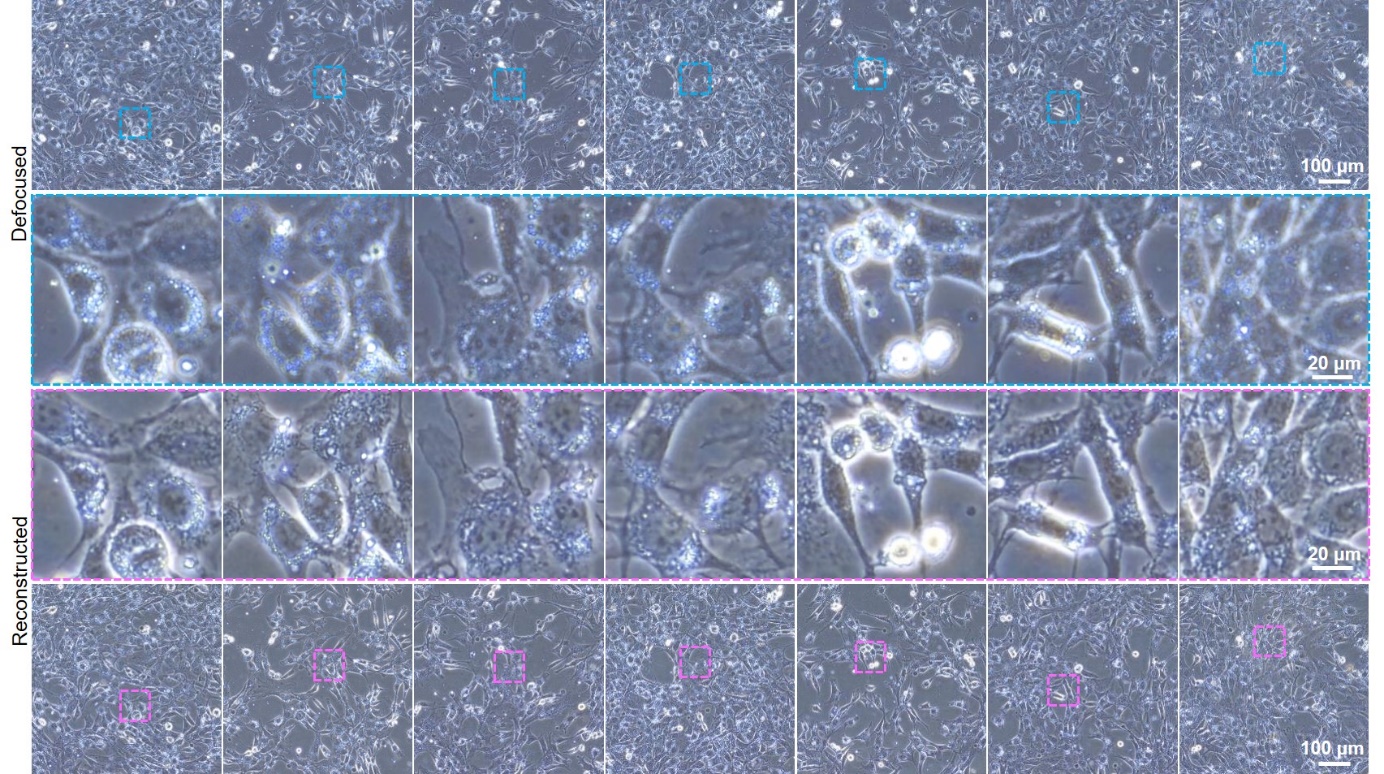


**Fig. S14.** The rest of the defocused cell images captured from the high-throughput chip and the corresponding reconstructed images. (Related to Fig. 1 & 9 in the main text).

**Table S1.** The number of images prepared for different experiments. ±10 here represents two values of +10 & –10 instead of all values within the range. ±20 & ±5 are the same.

| Cell Type | Use | Resolution | Magnification | Defocus Distance | Training Set | Validation Set | Testing Set |
| --- | --- | --- | --- | --- | --- | --- | --- |
| EC | Sorting | 536×536 px^2^ | 100×, 200× | N/A | 7560 | 1080 | 2160 |
| SMC |  |  |  | N/A | 3780 | 540 | 1080 |
| 3T3 |  |  |  | N/A | 3780 | 540 | 1080 |
|  | Reconstruction | 388×388 px^2^ | 100× | ±10 μm | 2712 | 340 | 340 |
|  |  |  |  | ±20 μm | 2712 | 340 | 340 |
|  |  |  | 200× | ±5 μm | 2944 | 368 | 368 |
|  |  |  |  | ±10 μm | 3072 | 384 | 384 |

**Table S2.** Parameters for the RL deconvolution. Related to Fig. S9.

| Channel | Filter Size | S.D. | Iteration | Defocus Distance |
| --- | --- | --- | --- | --- |
| R | [7×7] | 2 | 30 | -10 μm |
| G | [7×7] | 2 | 30 |  |
| B | [7×7] | 2 | 30 |  |
| R | [12×12] | 3.5 | 100 | -20 μm |
| G | [12×12] | 3.5 | 100 |  |
| B | [7×7] | 5 | 50 |  |
